# Supplementary material for: Highly active, ultra-low loading single-atom iron catalysts for catalytic transfer hydrogenation
Source: Nat Commun. 2023 Oct 20;14:6666. doi: 10.1038/s41467-023-42337-9 (PMC10589291; doi:10.1038/s41467-023-42337-9)
Supplement: Supplementary file 1 — Supplementary Information [file 41467_2023_42337_MOESM1_ESM.pdf]

Supporting information for

Highly Active, Ultra-Low Loading Single-Atom Iron Catalysts for Catalytic Transfer Hydrogenation

Zhidong An[1, ∇], Piaoping Yang[2, ∇], Delong Duan[3, ∇], Jiang Li[1, *], Tong Wan[1], Yue Kong[1], Stavros Caratzoulas[2], Shuting Xiang[4], Jiaxing Liu[1], Lei Huang[1], Anatoly I. Frenkel[4], Yuan-Ye Jiang[5], Ran Long[3, *], Zhenxing Li[1, *], and Dionisios G. Vlachos[2, *]

1. College of New Energy and Materials, China University of Petroleum (Beijing), Beijing, 102249, China.
2. Department of Chemical and Biomolecular Engineering and Catalysis Center for Energy Innovation, University of Delaware, 221 Academy St., Newark, Delaware, 19716, United States.
3. School of Chemistry and Materials Science, Frontiers Science Center for Planetary Exploration and Emerging Technologies, and National Synchrotron Radiation Laboratory, University of Science and Technology of China, Hefei, Anhui, 230026, China.
4. Department of Materials Science and Chemical Engineering, Stony Brook University, Stony Brook, New York, 11794, USA.
5. School of Chemistry and Chemical Engineering, Qufu Normal University, Qufu, 273165, China.

[*]Corresponding authors: lijiang@cup.edu.cn (Jiang Li), longran@ustc.edu.cn (Ran Long), lizx@cup.edu.cn (Zhenxing Li), vlachos@udel.edu. (Dionisios G. Vlachos).

[∇]These authors contributed equally.

Table of Contents

Figure S1. Experimental and simulation X-ray absorption spectra and analysis for the one-/two-step Fe-ZIF-8-800 catalysts. - 1 -
Figure S2. TEM images of ZIF-8 precursor. - 2 -
Figure S3. XRD pattern of ZIF-derived iron catalysts prepared at different pyrolysis temperatures. - 3 -
Figure S4. TGA data for Fe-ZIF-8 precursor. - 4 -
Figure S5. N_2 adsorption isotherms of various MOF-derived catalysts. - 5 -
Figure S6. O 1s XPS spectra of various MOF-derived catalysts. - 6 -
Figure S7. C 1s XPS spectra of various MOF-derived catalysts. - 7 -
Figure S8. Two representative aberration corrected HAADF-STEM images of Fe-ZIF-8-800 catalyst. - 8 -
Figure S9. Fe 2p XPS spectra of Fe-ZIF-8-800 catalyst. - 9 -
Figure S10. Experimental and simulation X-ray absorption spectra and analysis for the Fe-MOF-5-800 catalyst. - 10 -
Figure S11. EXAFS fitting of the Fe-ZIF-8-800 using Fe(II)-N_4C_{12} moiety... - 11 -
Figure S12. Comparison between the experimental K-edge XANES spectra and the theoretical spectra. - 12 -
Figure S13. Comparison between the experimental K-edge XANES spectra and the theoretical spectra. - 13 -
Figure S14. Evaluation of acid-base properties. - 15 -
Figure S15. Time course of FF conversion over Fe-ZIF-8-800 at 80 °C and 120 °C in 60 min. - 16 -
Figure S16. Time courses for the CTH of FF over Fe-ZIF-8-800 and Fe-phen/C-800 catalysts at different temperatures. - 17 -
Figure S17. Fe(II)-plN_3, Fe(II)-plN_4, and Zn(II)-plN_4 models. - 18 -
Figure S18. Optimized geometries (side and top views) of intermediates and transition states on P1 pathway. - 19 -
Figure S19. Optimized geometries (side and top views) of intermediates and transition states on P2 pathway. - 20 -
Figure S20. Optimized geometries (side and top views) of intermediates and transition states on P3 pathway for Fe(II)-plN_3. - 21 -
Figure S21. Gibbs free energy profiles along the P1 and P2 pathways for Fe(II)-plN_3 at 393 K. - 22 -
Figure S22. Crystal field splitting diagrams for various configurations of Fe(II) complexes. - 23 -
Figure S23. The adsorption geometries (side view) and corresponding adsorption energies of –OH groups in HMF and isopropanol at Fe(II)-plN_3. - 24 -
Figure S24. Catalyst stability benchmarking. - 25 -
Figure S25. Experimental X-ray absorption spectra for fresh and/or used Fe-ZIF-8-800 - 26 -
Figure S26. Experimental and computed K-edge XANES spectra. - 27 -
Figure S27. A representative GC spectrum that using benzyl alcohol as the internal standard. - 28 -

Figure S28. Corrected reaction formulas for pK_a prediction. .... - 29 -
Table S1. Contents of Fe and Zn in various MOF-derived catalysts. .... - 30 -
Table S2. Contents of Fe in Fe-ZIF catalysts prepared via the two-step route.- 31 -
Table S3. CTH performance of FF over one- and two-step Fe-ZIF-8-800 catalysts. .... - 32 -
Table S4. Fitting results of Fe K-edge EXAFS data of the one- and two-step Fe-ZIF-8-800 catalysts. .... - 33 -
Table S5. Contents of N, C and H in Fe-ZIF-8-800 catalyst. .... - 34 -
Table S6. Intensity ratio of D-band to G-band in Raman spectra of various MOF-derived catalysts. .... - 35 -
Table S7. N_2 adsorption/desorption data of various catalysts. .... - 36 -
Table S8. N speciation (at. %) from XPS analysis for various ZIF-derived catalysts. .... - 37 -
Table S9. O speciation (at. %) from XPS analysis for various MOF-derived catalysts. .... - 38 -
Table S10. C speciation (at. %) from XPS analysis for various MOF-derived catalysts. .... - 39 -
Table S11. Fitting results of Fe K-edge EXAFS data. .... - 40 -
Table S12. Fitting results of Zn K-edge EXAFS data. .... - 41 -
Table S13. Catalytic transfer hydrogenation of FF over various catalysts. ..... - 42 -
Table S14. Screening of H donor for the CTH of FF over Fe-ZIF-8-800 catalyst . - 44 -
Table S15. Representative examples of the conversion of FF to FA via catalytic transfer hydrogenation (CTH). .... - 45 -
Table S16. CTH of FF to FA over Fe-based catalysts. .... - 47 -
Table S17. Catalytic transfer hydrogenation of FF at various reaction conditions. - 49 -
Table S18. Bader charges of Fe and Zn sites for each state in Fe(II)-plN$_3$, Fe(II)-plN$_4$, Zn(II)-plN$_4$. .... - 50 -
Table S19. Theoretical values of turnover frequencies (TOFs) on Fe(II)-plN$_3$, Fe(II)-plN$_4$, and Zn(II)-plN$_4$. .... - 51 -
Table S20. The Fe and Zn contents in the reaction liquid after the first two reused cycle. The data were collected by ICP-AES analysis. .... - 52 -
Table S21. Fe contents and textural properties of fresh, reused, and regenerated Fe-ZIF-8-800 catalysts. .... - 53 -
Table S22. Cutting energy and core-level width for XANES simulation of Fe and Zn reference compounds. .... - 54 -
Table S23. Microkinetic model parameters. .... - 55 -
Table S24. Elementary steps and corresponding kinetic parameters on Fe(II)-plN$_3$, Fe(II)-plN$_4$, and Zn(II)-plN$_4$ (T=393 K). .... - 56 -
Table S25. Calculated relative solution-phase Gibbs free energies (in kcal/mol). . - 58 -
Table S26. Calculated thermodynamic corrections to Gibbs free energy (ΔG_{cor}), solution-phase single-point energies (ΔE_{sol}) and solution-phase Gibbs free

energies (ΔG_{sol}) in Hartree. .... - 59 -
Supplementary Note 1. Computational Details for pK_a prediction.................. - 60 -
References... - 62 -

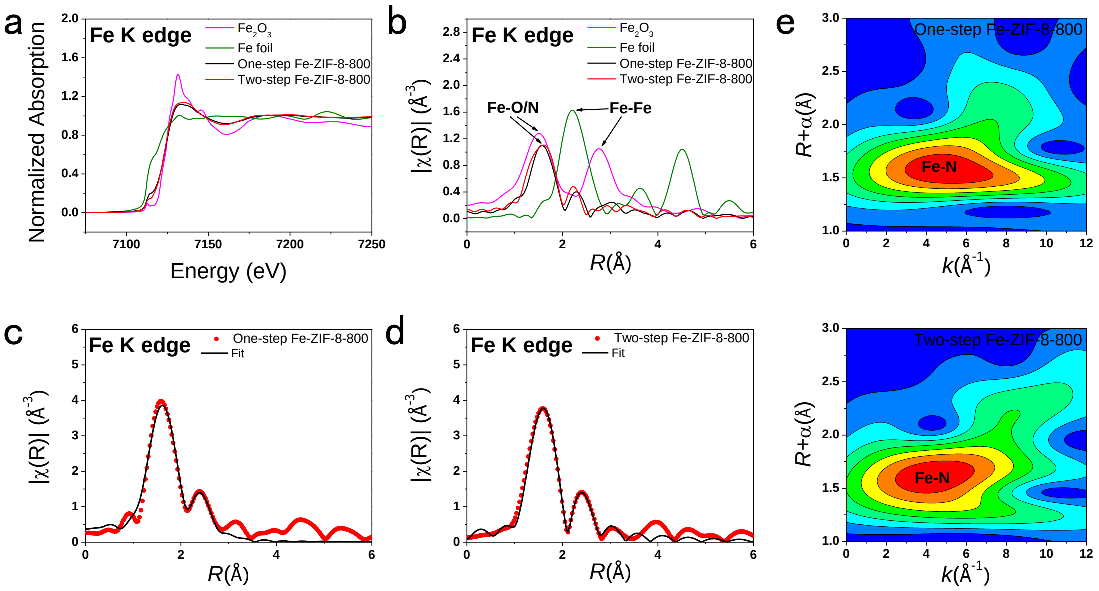

Figure S1. Experimental and simulation X-ray absorption spectra and analysis for the one-/two-step Fe-ZIF-8-800 catalysts. (a) Fe K-edge normalized X-ray absorption near-edge structure (XANES) spectra. (b) Fourier transform of k^2-weighted Fe K-edge EXAFS spectra. EXAFS fitting of the one-step (c) and two-step (d) Fe-ZIF-8-800 catalysts at Fe K-edge. (e) Wavelet transform of Fe K-edge EXAFS for one-step and two-step Fe-ZIF-8-800 catalysts.

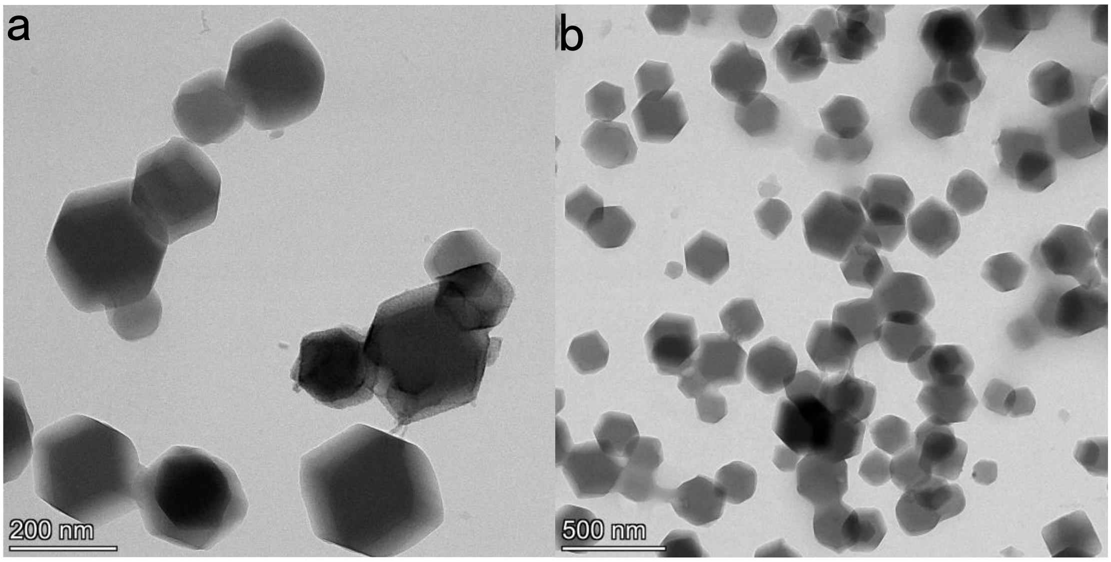

Figure S2. TEM images of ZIF-8 precursor. High-magnification (a) and low-magnification (b) TEM images of ZIF-8 precursor.

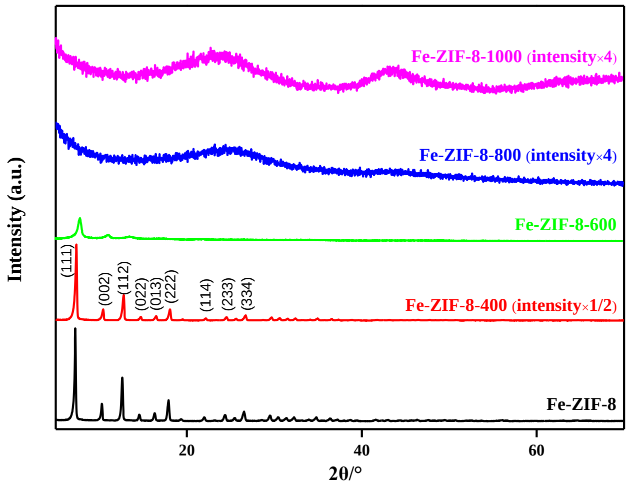

Figure S3. XRD pattern of ZIF-derived iron catalysts prepared at different pyrolysis temperatures.

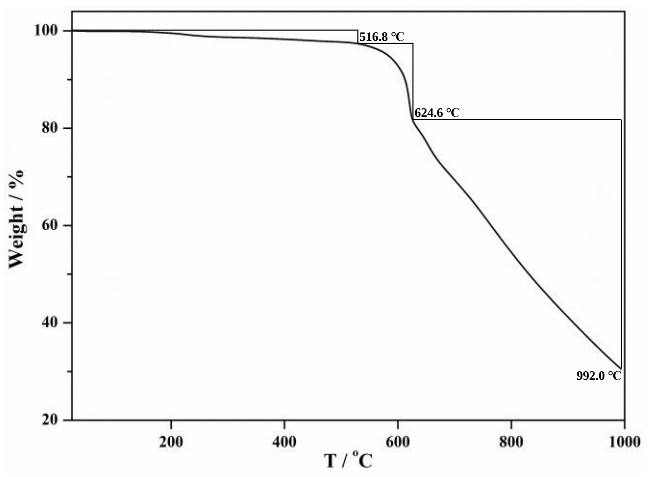

Figure S4. TGA data for Fe-ZIF-8 precursor.

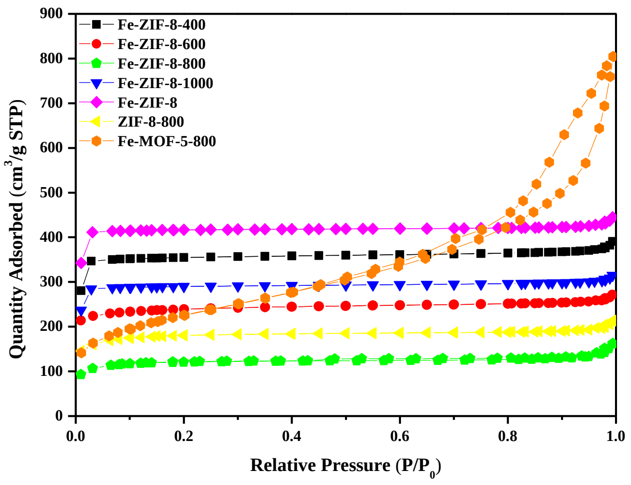

Figure S5. N_2 adsorption isotherms of various MOF-derived catalysts.

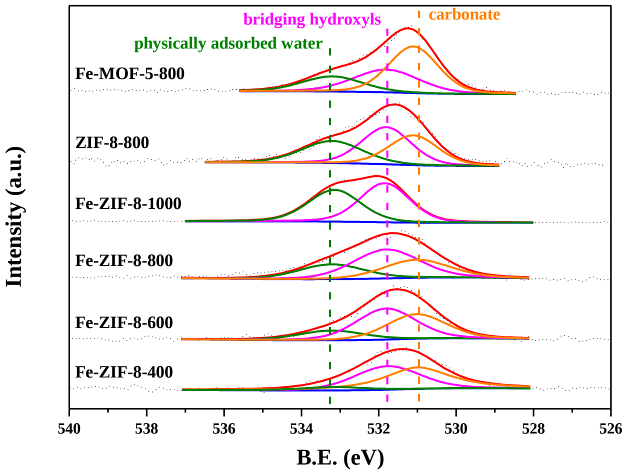

Figure S6. O 1s XPS spectra of various MOF-derived catalysts [S1,S2].

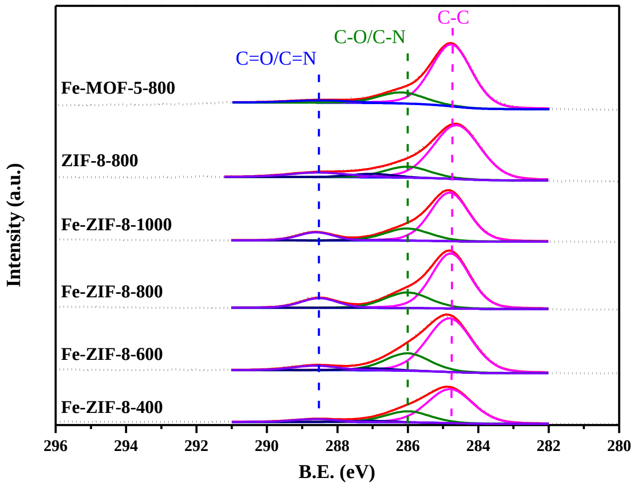

Figure S7. C 1s XPS spectra of various MOF-derived catalysts.

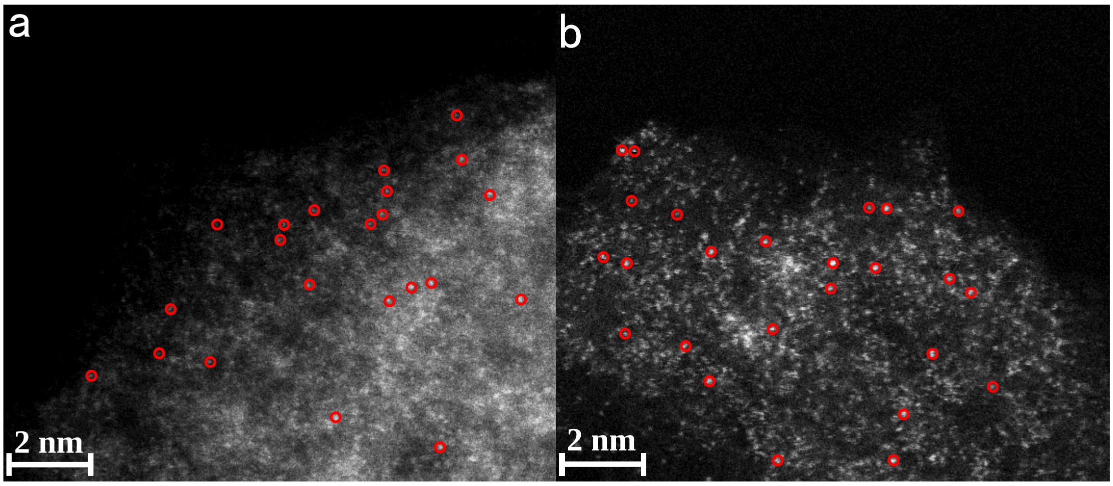

Figure S8. Two representative aberration corrected HAADF-STEM images of Fe-ZIF-8-800 catalyst. (a) and (b) represent different regions of Fe-ZIF-8-800 catalyst, respectively.

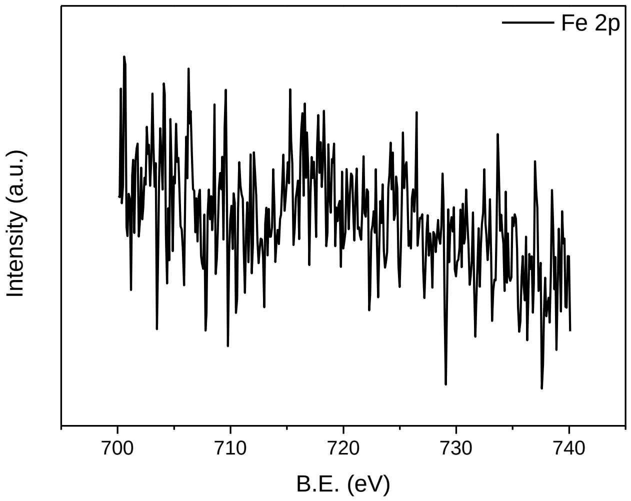

Figure S9. Fe 2p XPS spectra of Fe-ZIF-8-800 catalyst.

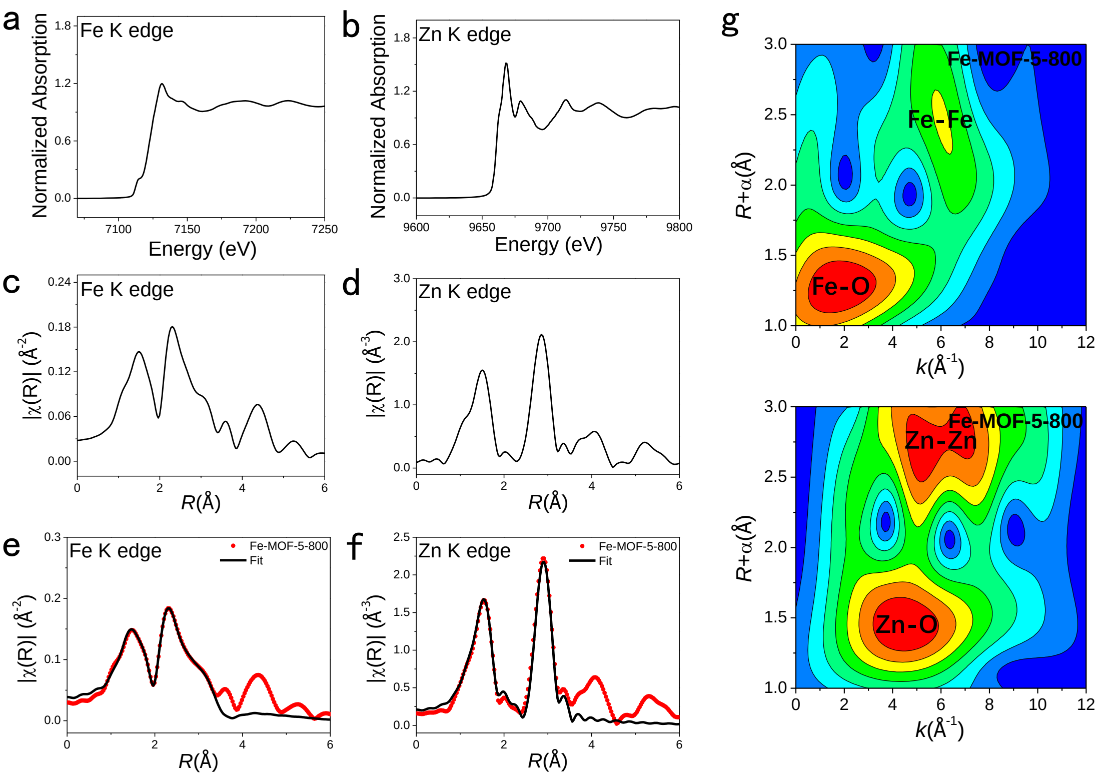

Figure S10. Experimental and simulation X-ray absorption spectra and analysis for the Fe-MOF-5-800 catalyst. Fe K-edge (a) and Zn K-edge (b) normalized X-ray absorption near-edge structure (XANES) spectra of the Fe-MOF-5-800 catalyst. Fourier transform of Fe K-edge (c) and Zn K-edge (d) EXAFS spectra of the Fe-MOF-5-800 catalyst. (e, f) EXAFS fitting of the Fe-MOF-5-800. (g) Wavelet transform of Fe K-edge and Zn K-edge EXAFS for Fe-MOF-5-800.

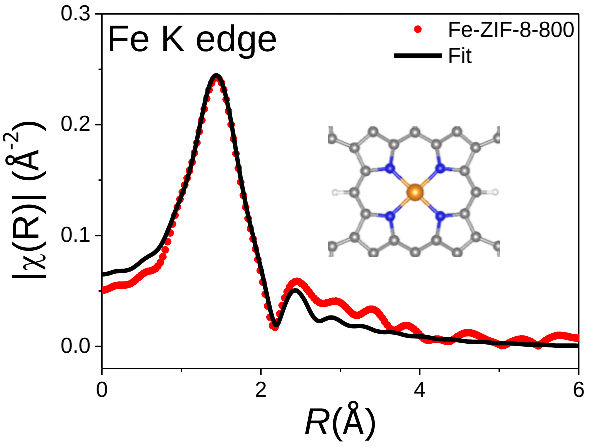

Figure S11. EXAFS fitting of the Fe-ZIF-8-800 using Fe(II)-N_4C_{12} moiety. The results are similar to that using Fe(II)-N_3C_{10} (**Figure 2**e), suggesting that the local structures of the Fe-N_x sites in Fe-ZIF-8-800 can be described well by both two porphyrin-based moieties. Insets: atomic structure models; Fe (orange), N (blue), and C (gray).

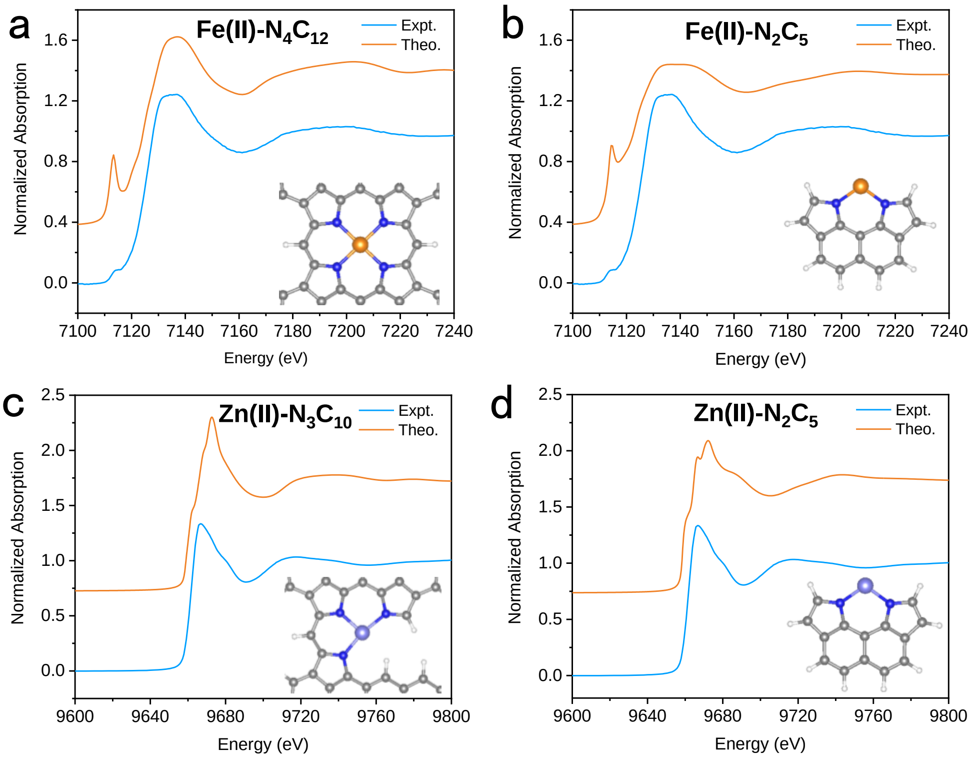

Figure S12. Comparison between the experimental K-edge XANES spectra and the theoretical spectra. (a–b) Experimental K-edge XANES spectra of Fe-ZIF-8-800 and theoretical spectra of the porphyrin-based Fe-N_xC_y moieties. (c–d) Experimental Zn K-edge XANES spectra of ZIF-8-800 and theoretical spectra of the porphyrin-based Zn-N_xC_y moieties. Latin numerals in brackets indicate the formal oxidation states of the metal atoms. Insets: atomic structure models; Fe (orange), Zn (light blue), N (blue), and C (gray).

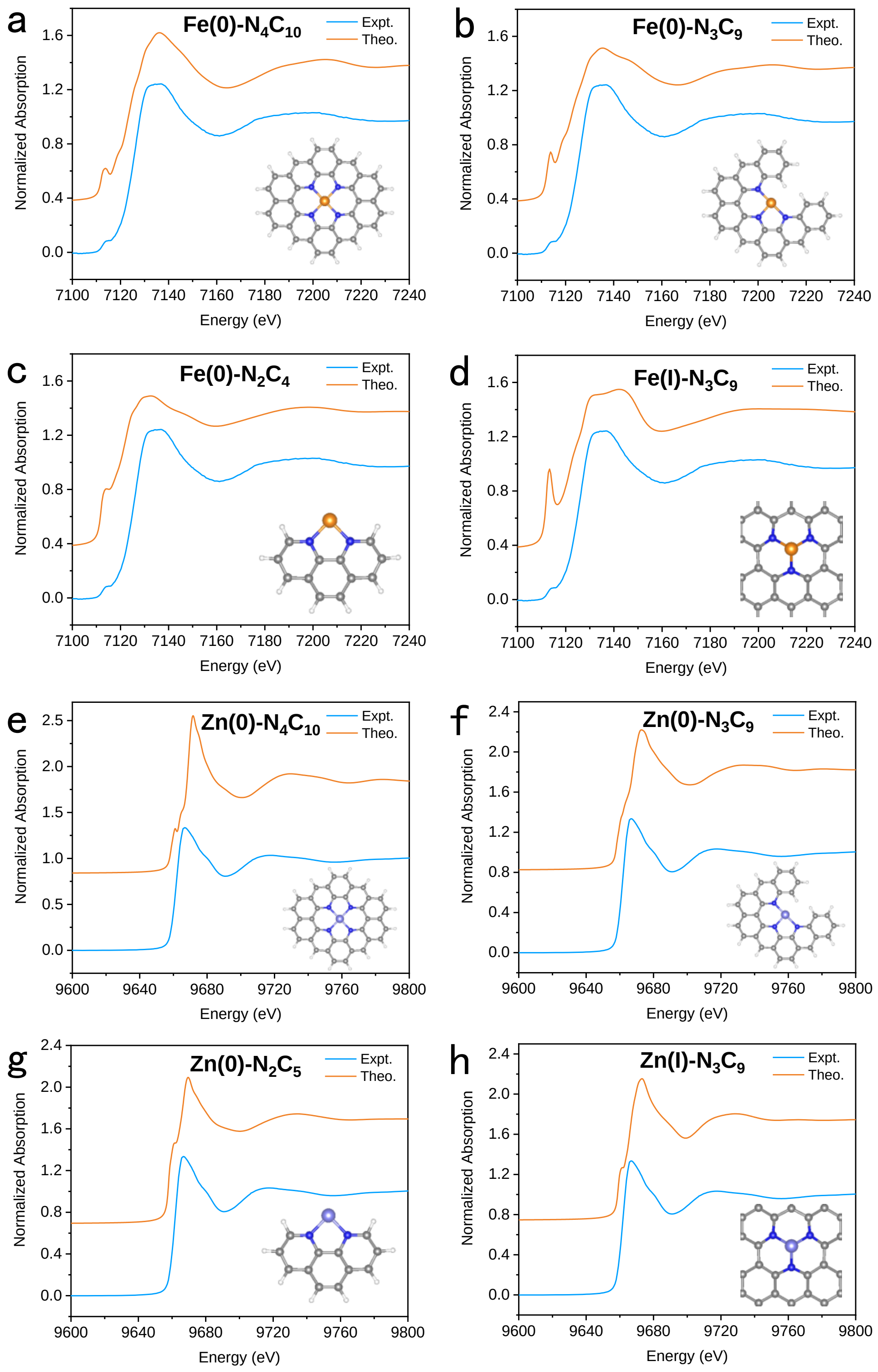

Figure S13. Comparison between the experimental K-edge XANES spectra and the theoretical spectra. (a–d) Experimental Fe K-edge XANES spectra of Fe-ZIF-8-800 and theoretical spectra of the Fe-N_xC_y moieties involving pyridinic N

coordination. (e–h) Experimental Zn K-edge XANES spectra of ZIF-8-800 and theoretical spectra of the Zn-N_xC_y moieties involving pyridinic N coordination. Latin numerals in brackets indicate the formal oxidation states of the metal atoms. Insets: atomic structure models; Fe (orange), Zn (light blue), N (blue), and C (gray).

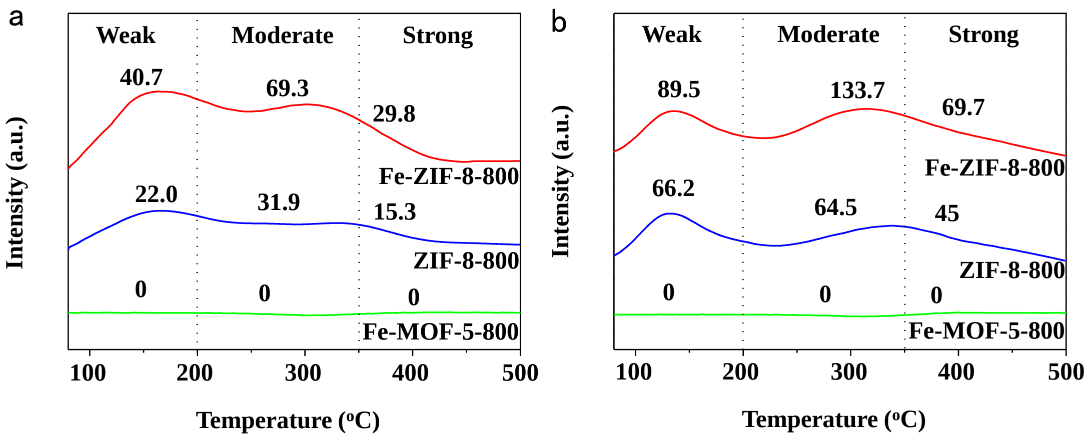

Figure S14. Evaluation of acid-base properties. NH_3-TPD (a) and CO_2-TPD (b) patterns of Fe-ZIF-8-800, ZIF-8-800, and Fe-MOF-5-800 catalysts.

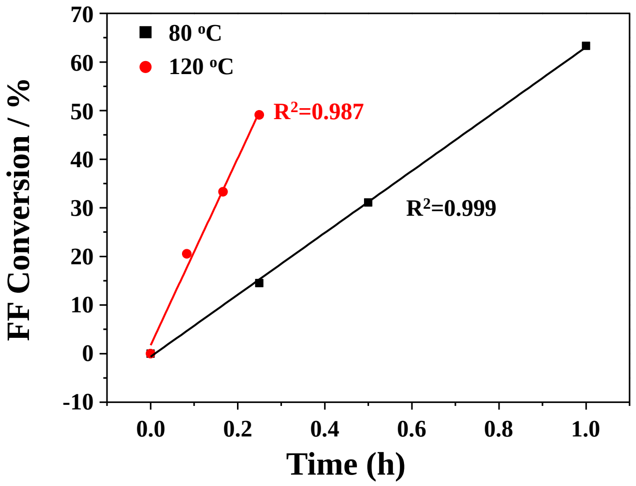

Figure S15. Time course of FF conversion over Fe-ZIF-8-800 at 80 ºC and 120 ºC in 60 min. Reaction conditions: 0.5 mmol FF, 3 mL solvent, 50 mg catalyst.

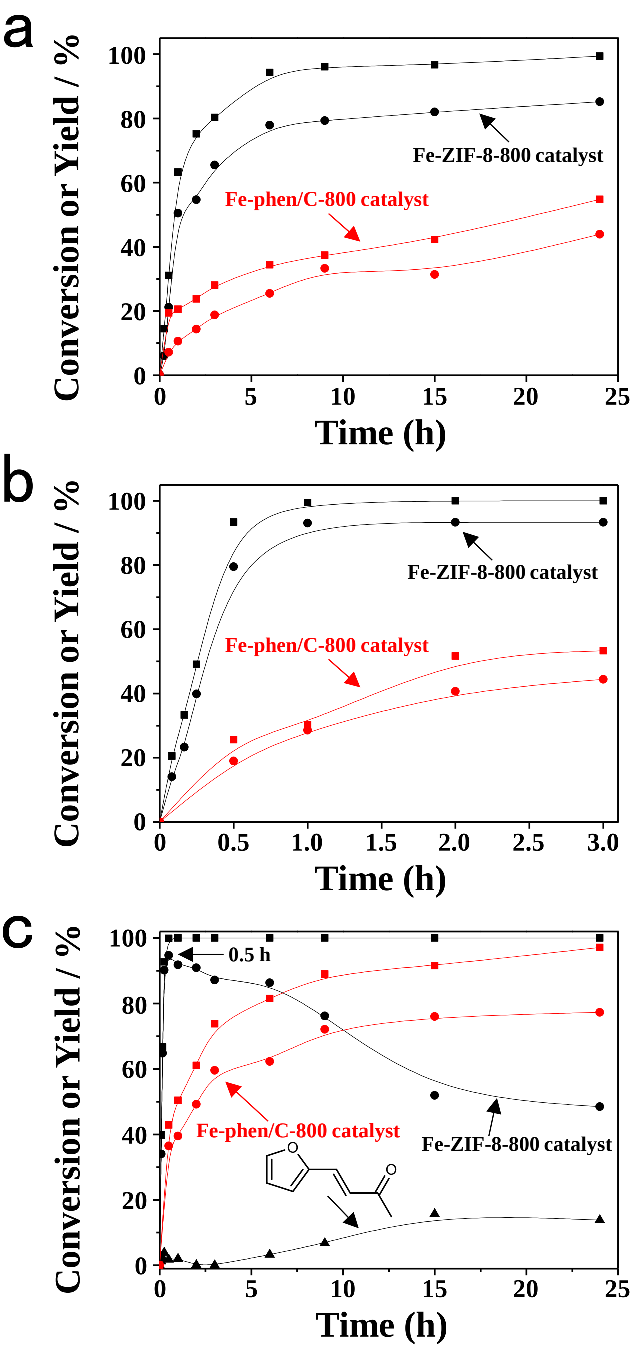

Figure S16. Time courses for the CTH of FF over Fe-ZIF-8-800 and Fe-phen/C-800 catalysts at different temperatures. 80 (a), 120 (b), and 160 °C (c) (squares: conversion, circles: yield). Reaction conditions: 0.5 mmol FF, 3 mL solvent, 50 mg catalyst.

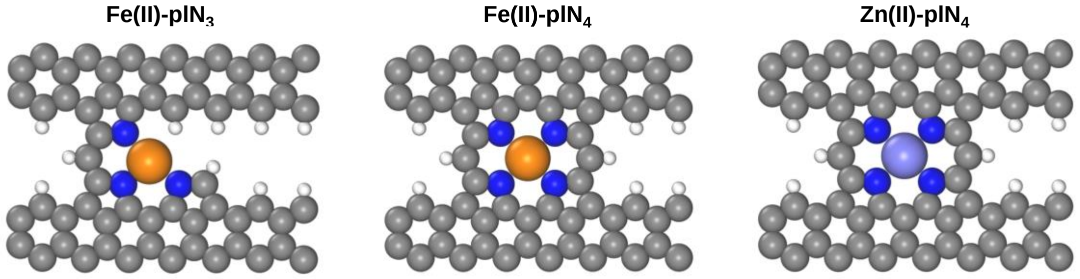

Figure S17. Fe(II)-plN$_3$, Fe(II)-plN$_4$, and Zn(II)-plN$_4$ models. The grey, blue, white, orange, and light purple spheres represent carbon (C), nitrogen (N), hydrogen (H), iron (Fe), and zinc (Zn), respectively.

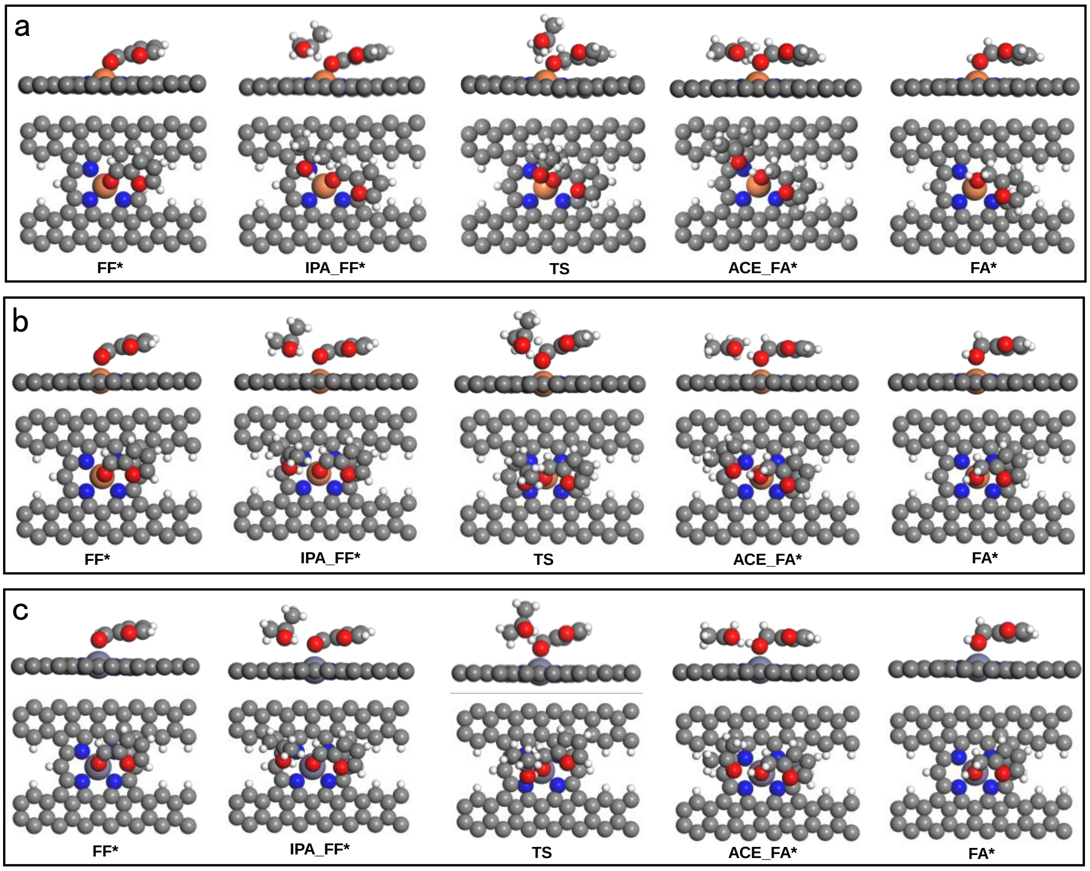

Figure S18. Optimized geometries (side and top views) of intermediates and transition states on P1 pathway. (a) Fe(II)-plN_3, Fe(II)-plN_4, and (c) Zn(II)-plN_4. The red, grey, blue, white, orange, and light purple spheres represent oxygen (O), carbon (C), nitrogen (N), hydrogen (H), iron (Fe), and zinc (Zn), respectively.

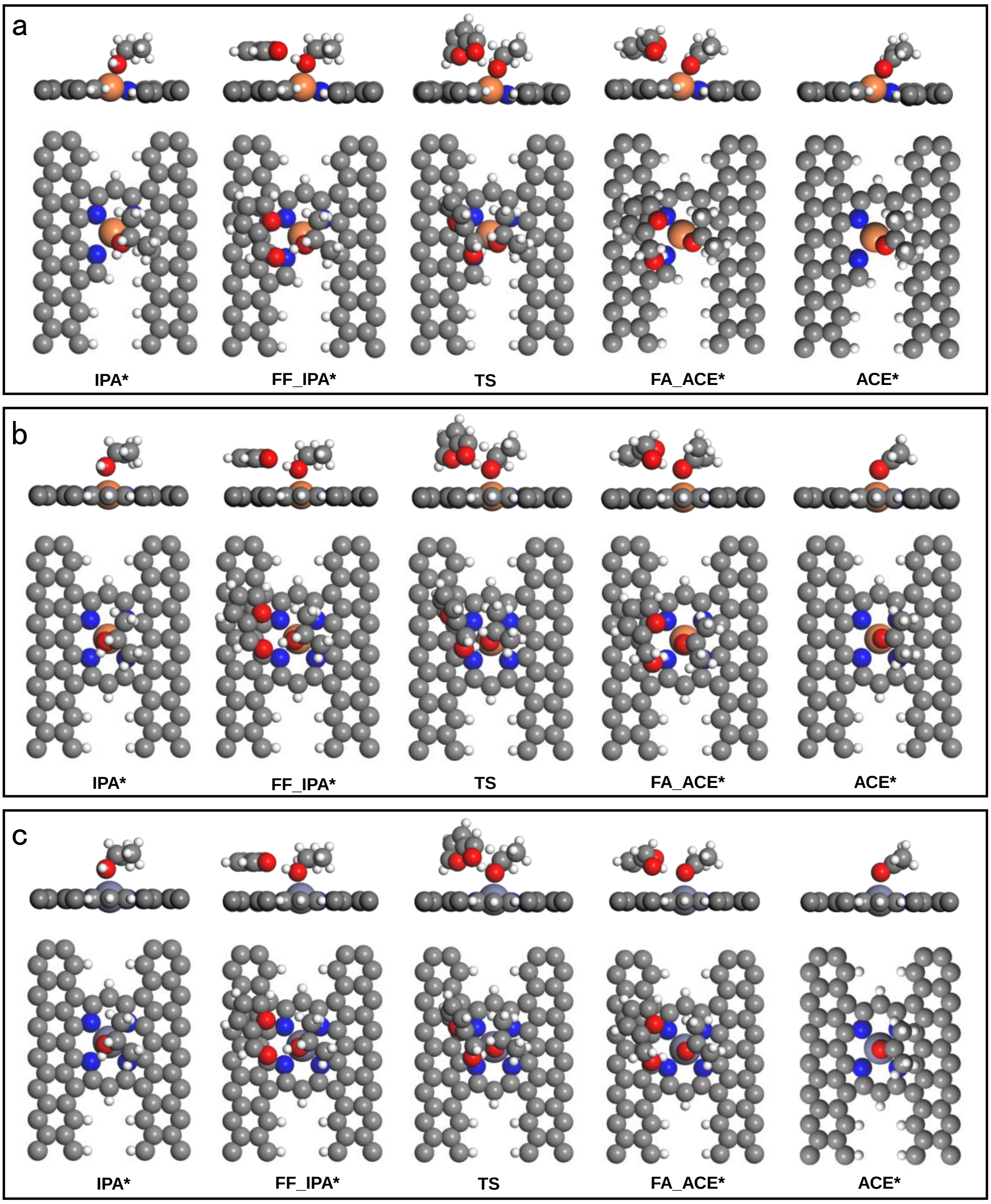

Figure S19. Optimized geometries (side and top views) of intermediates and transition states on P2 pathway. (a) Fe(II)-plN$_3$, (b) Fe(II)-plN$_4$, and (c) Zn(II)-plN$_4$. The red, grey, blue, white, orange, and light purple spheres represent oxygen (O), carbon (C), nitrogen (N), hydrogen (H), iron (Fe), and zinc (Zn), respectively.

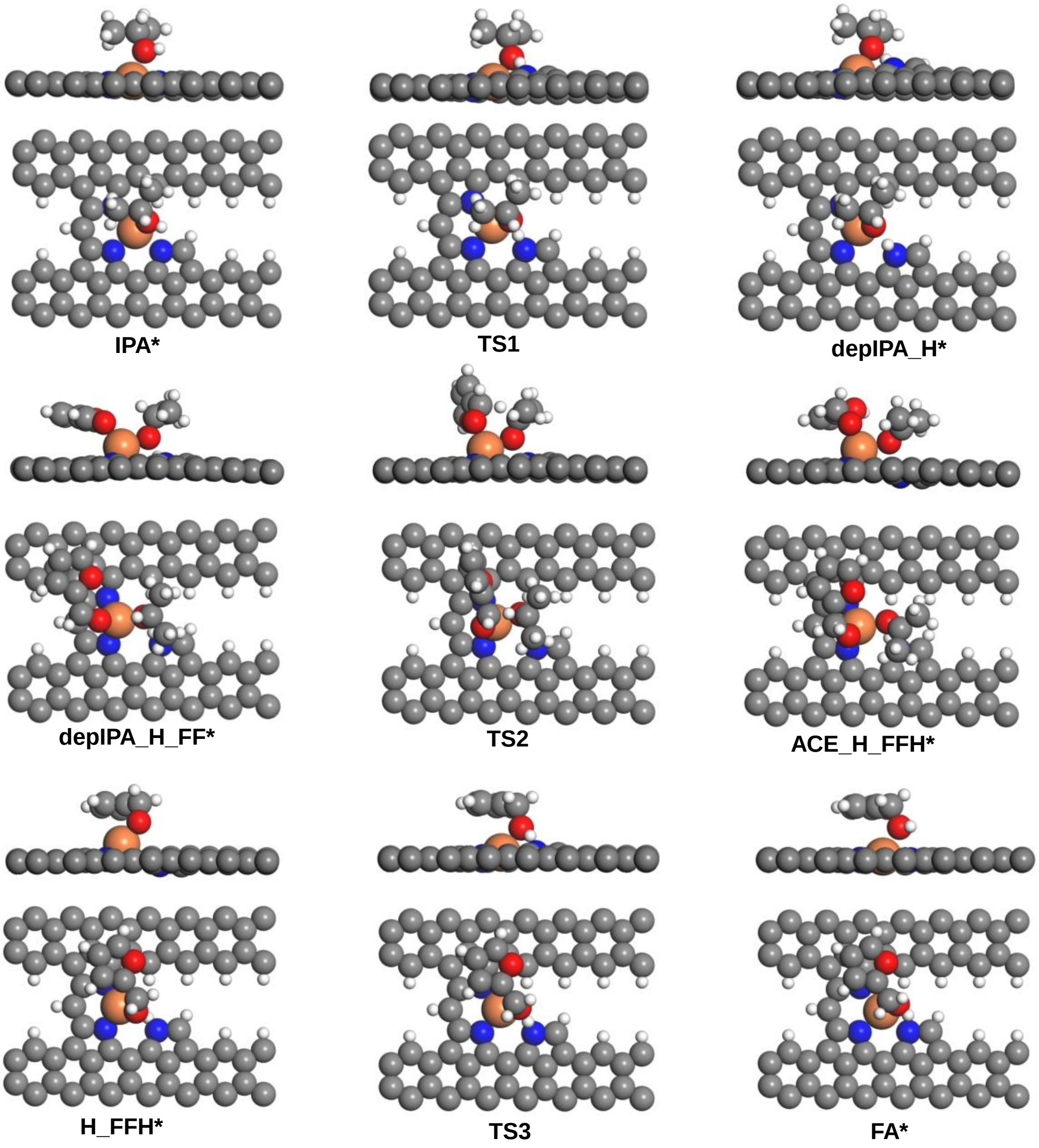

Figure S20. Optimized geometries (side and top views) of intermediates and transition states on P3 pathway for Fe(II)-plN₃. The red, grey, blue, white, orange, and light purple spheres represent oxygen (O), carbon (C), nitrogen (N), hydrogen (H), iron (Fe), and zinc (Zn), respectively.

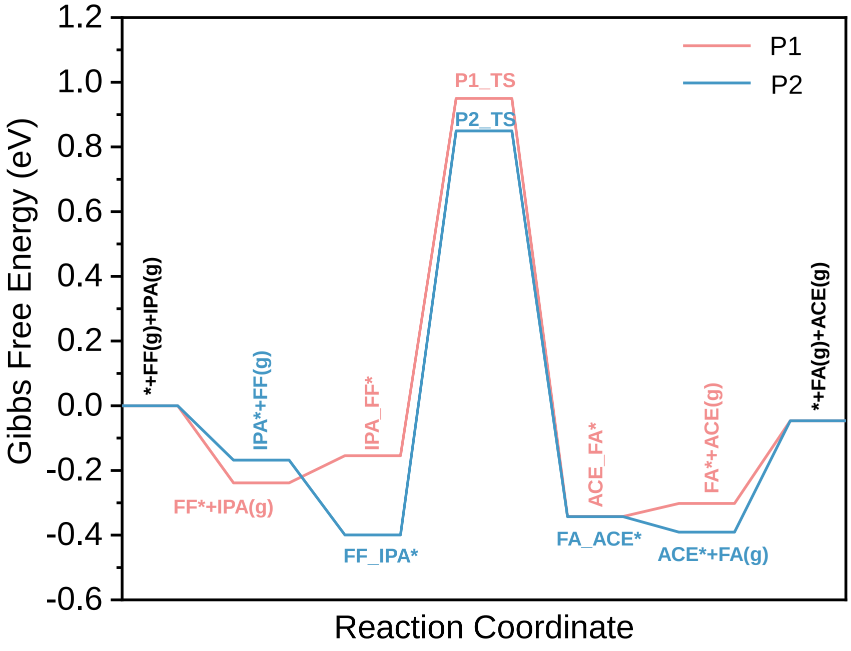

Figure S21. Gibbs free energy profiles along the P1 and P2 pathways for Fe(II)-plN_3 at 393 K.

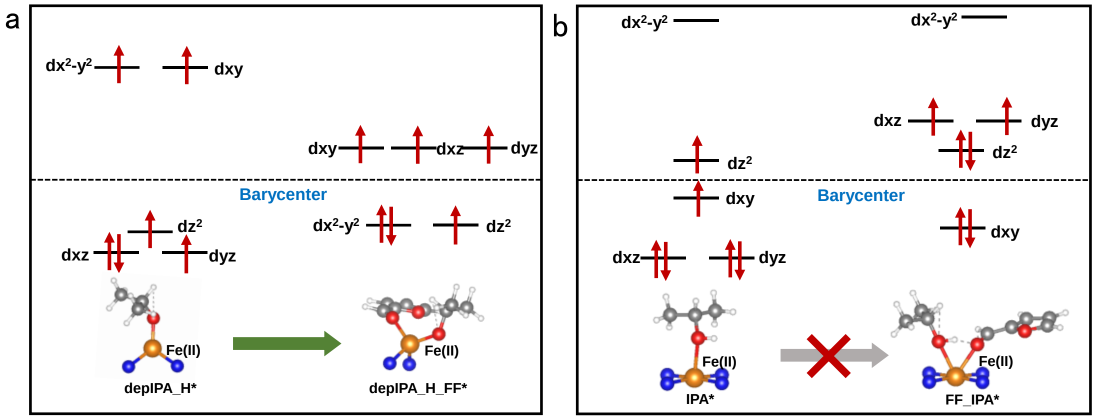

Figure S22. Crystal field splitting diagrams for various configurations of Fe(II) complexes. (a) Trigonal configuration to tetrahedral configuration, (b) Square pyramidal configuration to ridge-tent configuration. depIPA_H*, depIPA_H_FF*, IPA*, FF_IPA* denote the trigonal, tetrahedral, square pyramidal, and “ridge-tent” configurations, respectively. The red, grey, blue, white, and orange spheres represent oxygen (O), carbon (C), nitrogen (N), hydrogen (H), and iron (Fe), respectively.

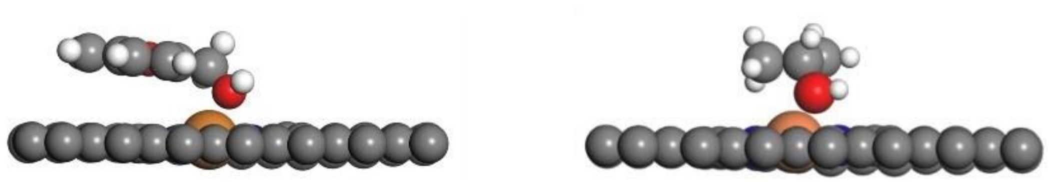

HMF_OH* (-1.05 eV) **IPA* (-0.93 eV)**

Figure S23. The adsorption geometries (side view) and corresponding adsorption energies of –OH groups in HMF and isopropanol at Fe(II)-plN$_3$. The red, grey, blue, white, and orange spheres represent oxygen (O), carbon (C), nitrogen (N), hydrogen (H), and iron (Fe), respectively.

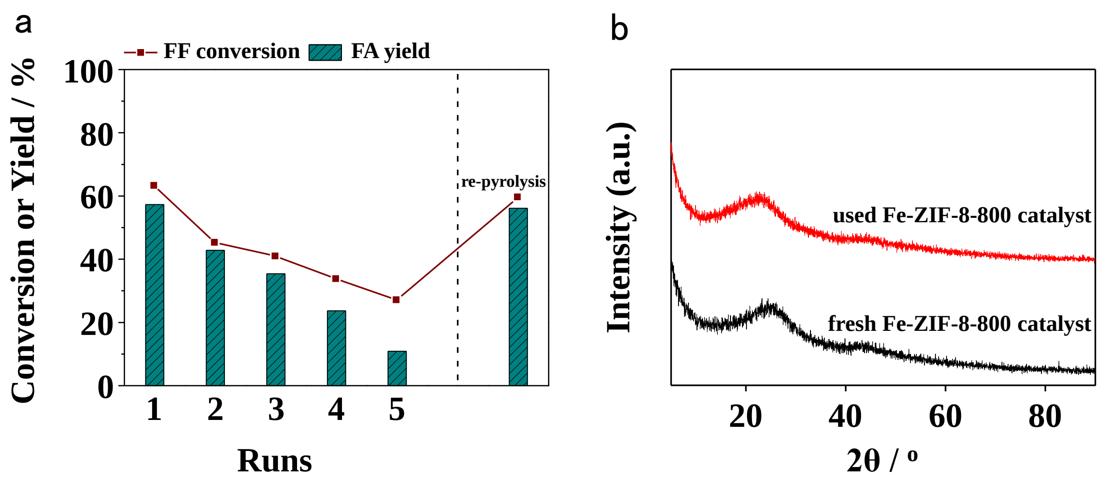

Figure S24. Catalyst stability benchmarking. (a) Recyclability tests of Fe-ZIF-8-800 catalyst. Reaction conditions: 0.5 mmol FF, 3 mL isopropanol solvent, 120 °C, 15 min, 50 mg Fe-ZIF-8-800 catalyst for the first run, and then reused for 4 runs. To recover the activity of Fe-ZIF-8-800 catalyst, re-pyrolysis of the reused catalyst is proceeded by adding 0.033 wt% $Fe(NO_3)_3$ (the Fe dosage is only 0.01 wt%). (b) XRD pattern of used and fresh Fe-ZIF-8-800 catalysts.

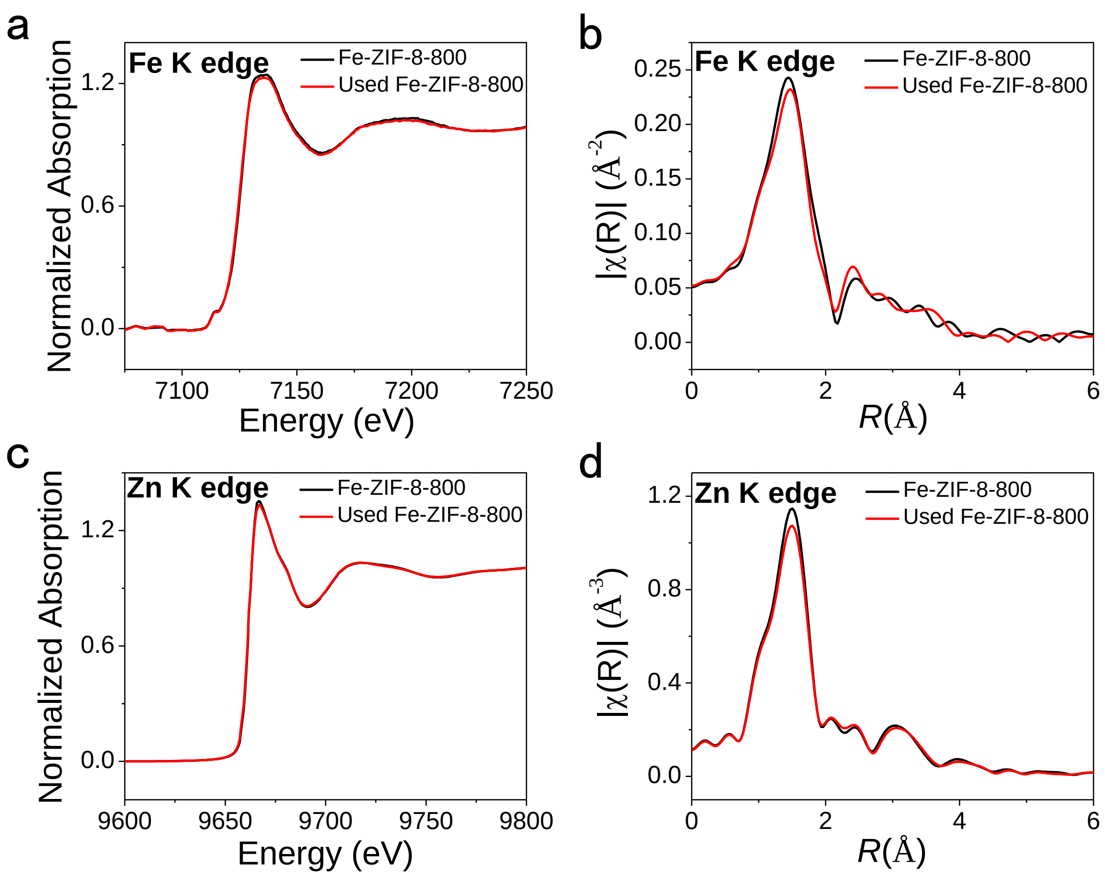

Figure S25. Experimental X-ray absorption spectra for fresh and/or used Fe-ZIF-8-800. Fe K-edge (a) and Zn K-edge (c) normalized XANES spectra of fresh and used Fe-ZIF-8-800 catalysts. Fourier transform of Fe K-edge (b) and Zn K-edge (d) EXAFS spectra of fresh and used Fe-ZIF-8-800 catalysts.

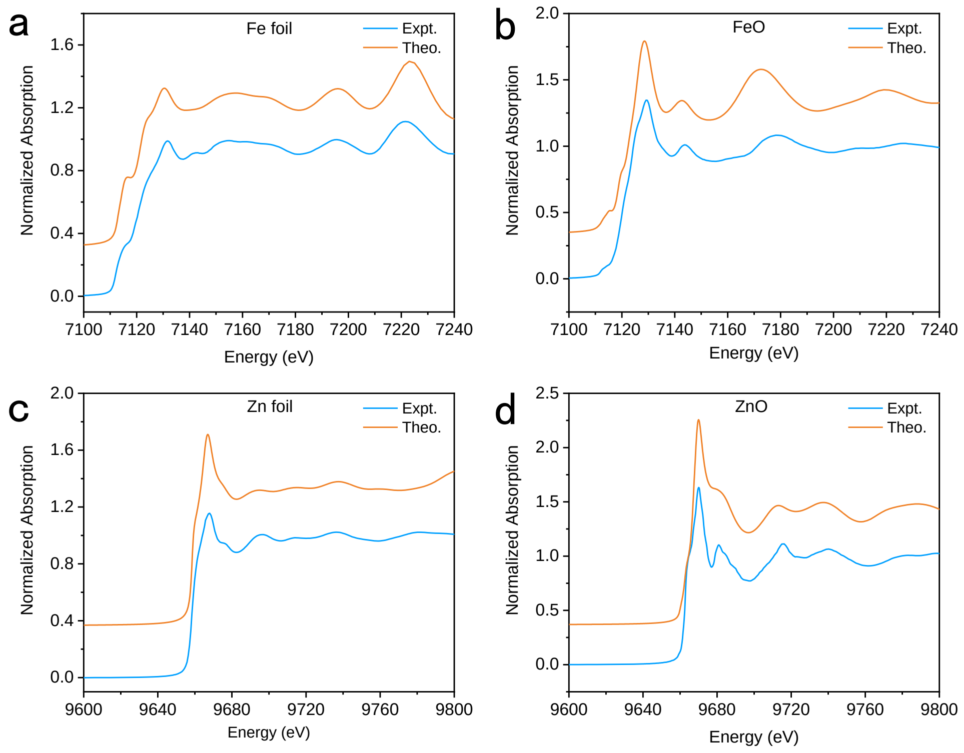

Figure S26. Experimental and computed K-edge XANES spectra. Fe foil (a), FeO (b), Zn foil (c), and ZnO (d).

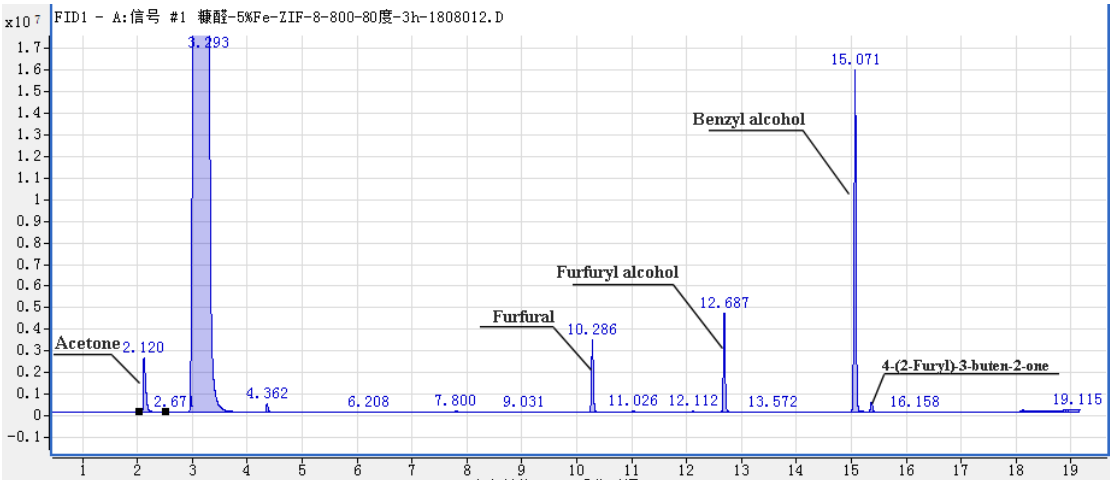

Figure S27. A representative GC spectrum that using benzyl alcohol as the internal standard. Reaction conditions: 0.5 mmol FF, 3 mL isopropanol solvent, 50 mg Fe-ZIF-8-800 catalyst (0.13 mol% Fe), 80 ºC, 3 h.

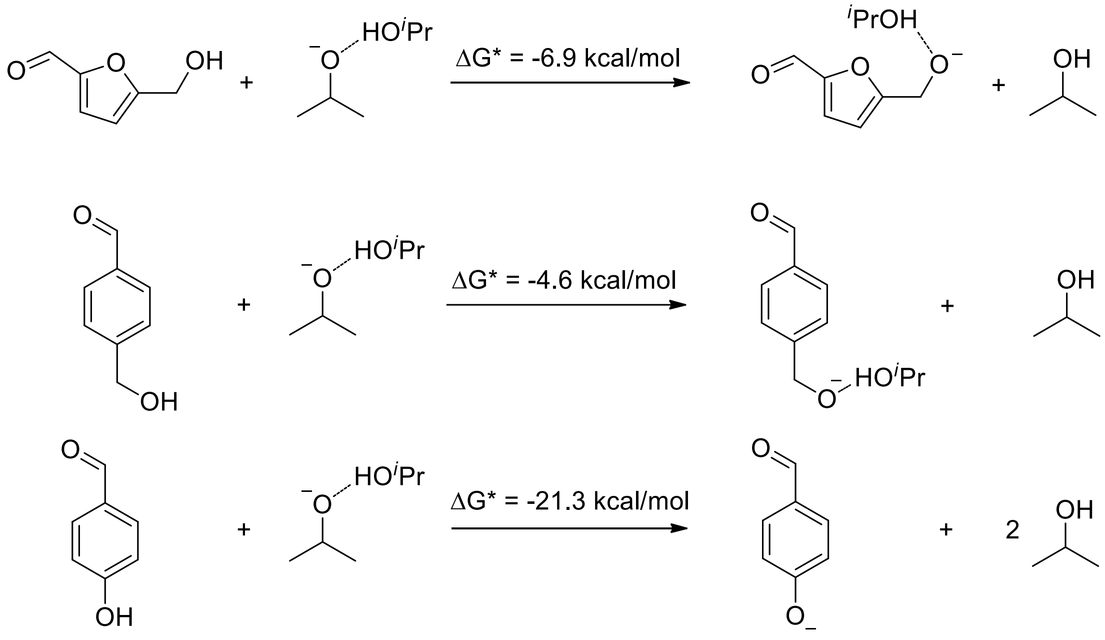

Figure S28. Corrected reaction formulas for pK_a prediction.

Table S1. Contents of Fe and Zn in various MOF-derived catalysts.

Entry	Catalyst	Fe Content [%]	Zn Content [%]
1	Fe-ZIF-8-800	0.073	28.60
2	Fe-MOF-5-800	5.73	63.24
3	ZIF-8	–	30.81
4	ZIF-8-800	–	20.79
5	Fe-ZIF-8	0.037	30.48
6	Fe-MOF-5	0.33	N.D.
7	Fe-ZIF-8-400	N.D.	30.95
8	Fe-ZIF-8-600	N.D.	33.29
9	Fe-ZIF-8-1000	N.D.	1.41

N.D.=not determined.

Table S2. Contents of Fe in Fe-ZIF catalysts prepared via the two-step route.

Entry	Catalyst	Fe Content [%]
1	0.1%Fe-ZIF-8-800	0.08
2	0.3%Fe-ZIF-8-800	0.29

Table S3. CTH performance of FF over one- and two-step Fe-ZIF-8-800 catalysts[a].

Entry	Catalyst	T [ºC]	T [min]	Conv. [%]	Yield [%]	TOF [h^{-1}]
1	Fe-ZIF-8-800[b]	80	30	24.0	17.6	367.2
2		120	360	99.6	96.5	1881.9[c]
3	Fe-ZIF-8-800[d]	80	30	23.8	13.8	320.7
4		120	360	96.7	93.8	1843.1[c]

[a]Reaction conditions: 0.5 mmol FF, 3 mL isopropanol solvent, 50 mg catalyst. [b]Prepared via the one-step route. [c]TOF was calculated based on the FF conversion at 5 min. [d]Prepared via the two-step route.

Table S4. Fitting results of Fe K-edge EXAFS data of the one- and two-step Fe-ZIF-8-800 catalysts.

Sample	Scattering Path	CN	R(Å)
Fe-ZIF-8-800[a]	Fe-N	3.5 ± 0.7	1.97 ± 0.04
Fe-ZIF-8-800[b]	Fe-N	3.6 ± 0.5	1.97 ± 0.05

[a]One-step Fe-ZIF-8-800 catalyst; [b]two-step Fe-ZIF-8-800 catalyst; CN, the coordination number; R, the bonding distance.

Table S5. Contents of N, C and H in Fe-ZIF-8-800 catalyst.

Catalyst	N [wt%]	C [wt%]	H [wt%]
Fe-ZIF-8-800	21.98	46.34	2.374

Table S6. Intensity ratio of D-band to G-band in Raman spectra of various MOF-derived catalysts.

Catalyst	I_D/I_G	Peak Intensity	
		D peak	G peak
Fe-ZIF-8-800	2.01	7.66	3.82
Fe-ZIF-8-1000	1.60	7.43	4.64
Fe-MOF-5-800	1.03	5.71	5.55
ZIF-8-800	1.38	15.03	10.89

Table S7. N_2 adsorption/desorption data of various catalysts.

Catalyst	BET Surface Area (m^2/g)	Pore Size (nm)	Pore Volume (cm^3/g)
Fe-ZIF-8	1315	9.62	0.05
Fe-ZIF-8-400	1280	7.53	0.07
Fe-ZIF-8-600	766	5.00	0.07
Fe-ZIF-8-800	403	9.75	0.07
Fe-ZIF-8-1000	991	7.83	0.05
ZIF-8-800	580	6.64	0.07
Fe-MOF-5-800	798	7.74	1.09

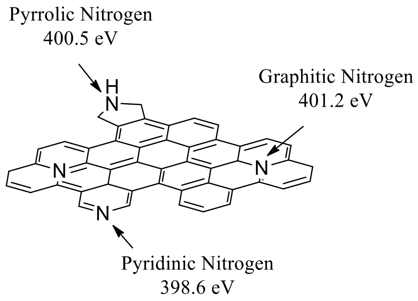

N-doped carbon

Table S8. N speciation (at. %) from XPS analysis for various ZIF-derived catalysts.

Sample	pyridnic N 398.6 eV	pyrrolic N 400.5 eV	graphitic N 401.2 eV
Fe-ZIF-8-400	93.3	6.7	0
Fe-ZIF-8-600	90.4	9.6	0
Fe-ZIF-8-800	81.1	13.1	5.8
ZIF-8-800	72.8	18.5	8.7

Table S9. O speciation (at. %) from XPS analysis for various MOF-derived catalysts.

Sample	carbonate	bridging hydroxyls	physically adsorbed water	total O (at. %)
Fe-ZIF-8-400	3.6	3.1	0.4	7.1
Fe-ZIF-8-600	3.7	4.6	1.6	9.9
Fe-ZIF-8-800	2.3	3.7	1.9	7.9
Fe-ZIF-8-1000	0	11.5	9.9	21.4
Fe-MOF-5-800	6.4	4.1	2.5	13.0
ZIF-8-800	4.2	5.3	3.9	13.3

Table S10. C speciation (at. %) from XPS analysis for various MOF-derived catalysts.

Sample	C–C/C–H	C–N/C–O	C=O/C=N	O–C=O	total C (at. %)
Fe-ZIF-8-400	46.6	15.9	0	3.0	65.5
Fe-ZIF-8-600	45.5	14.8	0	3.8	64.1
Fe-ZIF-8-800	50.1	16.3	0	8.3	74.8
Fe-ZIF-8-1000	53.2	15.7	0	8.1	77.1
Fe-MOF-5-800	49.5	9.9	0	2.8	62.2
ZIF-8-800	55.0	9.3	4.9	6.4	75.7

Table S11. Fitting results of Fe K-edge EXAFS data.

Sample	Scattering Path	CN	R(Å)
Fe-ZIF-8-800	Fe-N	3.7 ± 0.6	1.96 ± 0.02
Used Fe-ZIF-8-800	Fe-N	4.6 ± 0.6	2.00 ± 0.10
Fe-MOF-5-800	Fe-O	4.8 ± 1.2	2.05 ± 0.09
	Fe-Fe	4.7 ± 2.3	2.47 ± 0.06
Fe_2O_3	Fe-O	6.0 (fixed)	1.94 ± 0.01
	Fe-Fe	1.0 (fixed)	2.70 ± 0.05
	Fe-Fe	3.0 (fixed)	2.96 ± 0.01
	Fe-Fe	3.0 (fixed)	3.45 ± 0.09
FeO	Fe-O	6.0 (fixed)	2.11 ± 0.06
	Fe-Fe	12.0 (fixed)	3.07 ± 0.01
Fe foil	Fe-Fe	8.0 (fixed)	2.47 ± 0.02
	Fe-Fe	6.0 (fixed)	2.85 ± 0.02

CN, the coordination numbers; R, the bonding distance.

Table S12. Fitting results of Zn K-edge EXAFS data.

Sample	Scattering Path	CN	R(Å)
Fe-ZIF-8-800	Zn-N	3.9 ± 0.3	1.97 ± 0.01
Used Fe-ZIF-8-800	Zn-N	4.1 ± 0.4	1.98 ± 0.01
Fe-MOF-5-800	Zn-O	4.6 ± 0.5	1.98 ± 0.17
	Zn-Zn	6.2 ± 2.6	3.22 ± 0.07
ZIF-8-800	Zn-N	4.1 ± 0.4	1.98 ± 0.01
ZnO	Zn-O	4.0 (fixed)	1.97 ± 0.00
	Zn-Zn	12.0 (fixed)	3.23 ± 0.02
Zn foil	Zn-Zn	12.0 (fixed)	2.65 ± 0.01

CN, the coordination numbers; R, the bonding distance.

Table S13. Catalytic transfer hydrogenation of FF over various catalysts.[a]

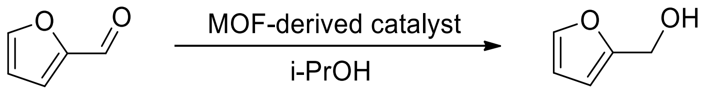

Entry	Catalyst	T [°C]	t [h]	Conv. [%]	Yield [%]	Sel. [%]	Yield of solvent-derived products	
							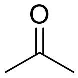	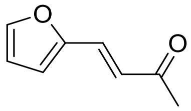
1	–	120	6	9.3	1.1	12.1	1.1	<0.1
2	Fe-ZIF-8-800	120	6	99.6	96.5	96.9	156.0	2.1
3	Fe-phen/C-800	120	6	51.5	44.0	85.4	87.9	0.3
4	Fe-MOF-5-800	120	6	11.4	3.3	28.9	139.4	0
5	ZIF-8-800	120	6	22.1	12.0	54.3	33.7	0.4
6	Fe-ZnO-NH_3-800[b]	120	6	4.3	1.2	27.9	2.3	0
7	$Fe(acac)_3$-ZIF-8-800[c]	120	6	95.0	74.0	77.9	112.1	4.8
8	$Fe(acac)_3$-ZIF-8-900[d]	120	6	51.4	37.0	72.0	67.6	0
9	Fe-ZIF-8	120	6	23.1	13.0	56.3	54.2	0.6
10	ZIF-8	120	6	14.8	5.7	38.5	22.7	0
11	$Fe(NO_3)_3$[e]	120	6	4.9	0.6	12.2	5.9	0
12	$Fe(acac)_3$[e]	120	6	11.8	3.0	25.4	3.6	<0.1
13	Fe-ZIF-8-400	120	6	4.8	2.2	45.8	3.14	0
14	Fe-ZIF-8-600	120	6	82.0	79.6	97.1	82.1	1.8
15	Fe-ZIF-8-900	120	6	98.5	87.0	88.3	125.7	2.8
16	Fe-ZIF-8-1000	120	6	61.8	50.1	81.1	110.3	1.6
17	Fe-ZIF-8-800	120	1	99.5	93.1	93.5	103.8	2.7
18[f]	Fe-ZIF-8-800	80	3	96.6	90.1	93.3	103.8	3.6
19	Pd/C	120	1	14.8	0.6	4.1	5.8	0
20	Pt/C	120	1	16.3	0.7	4.3	3.5	0
21	Ru/C	120	1	36.9	27.7	75.1	4.0	0

[a]Reaction conditions: 0.5 mmol FF, 3 mL isopropanol solvent, 50 mg catalyst (0.13 mol% Fe). [b]Calcination of Fe-ZnO precursor under NH_3 at 800 °C. The dosage of Fe in Fe-ZnO precursor is the same as Fe-ZIF-8-800 catalyst. [c]The synthesis method is the same as Li's paper [S3] except pyrolysis at 800 °C. [d]The synthesis method is the same as Li's paper [S3]. [e]The dosage of Fe is the same as Fe-ZIF-8-800 catalyst. [f]Iron loading is 0.3 wt% (0.54 mol% Fe).

The catalytic performance of various MOF-derived catalysts is summarized in

Table S13. The formation of acetone (dehydrogenation product of isopropanol solvent) and of (E)-4-(furan-2-yl)but-3-en-2-one (coupling products of FF and acetone) illustrates solvent conversion and side reaction, respectively. The higher yields of acetone than that of FA (entries 2–17) indicated that the catalysts have independent dehydrogenation ability, which was confirmed by the linear correlation between the Fe loading and the specific reaction rate of acetone (**Figure 3**b). Furthermore, the specific rate of acetone is *ca.* 5 times lower than that of FF or FA, indicating transfer hydrogenation is more dominant in our work than the dehydrogenation-hydrogenation route. Trace FA is observed in the blank test (entry 1). We also tried Li's method [S3] to prepare Fe SA catalysts using $Fe(acac)_3$ as Fe precursors. The performance is inferior to our Fe SA catalyst although the Fe loading is much higher (2.16 wt%, *ca.* 30 times higher than our Fe-ZIF-8-800 catalyst) in their work. Control experiments using noble metal catalysts such as Pd, Pt, and Ru are inferior to ZIF-derived catalysts (entries 16–18).

Table S14. **Screening of H donor for the CTH of FF over Fe-ZIF-8-800 catalyst.**[a]

Entry	H-Donor	Conversion [%]	Yield [%]	Selectivity [%]
1	MeOH	28.8	13.4	47.1
2	EtOH	42.1	33.7	80.0
3	1-PrOH	60.0	29.4	49.1
4	2-PrOH	99.6	96.5	96.9
5	2-BuOH	96.2	89.1	92.7
6	Cyclohexanol	49.3	25.7	52.1

[a]Reaction conditions: 0.5 mmol FF, 3 mL solvent, 50 mg Fe-ZIF-8-800 catalyst, 120 ^{o}C, 6 h.

Various alcohols are examined to explore the effect of H donor (**Table S14**). Primary alcohols, such as methanol, ethanol, and 1-propanol give FA yields of <35% at FF conversions of <60%. The conversion of FF increases with increasing carbon number. Secondary alcohols, such as 2-propanol and 2-butanol, furnish FA selectivities of >90% at excellent FF conversions of >95%. Cyclohexanol leads to inferior catalytic performance due to the high viscosity that prevents the efficient contact of the catalyst and the substrate. Overall, the results, along with the dehydrogenation of a fraction of the solvent, are consistent with the Meerwein-Ponndorf-Verley (MPV) concerted reaction mechanism [S4,S5].

Table S15. Representative examples of the conversion of FF to FA via catalytic transfer hydrogenation (CTH).

Entry	Catalyst	Solvent	T[a] [K]	P[b] [MPa]	t[c] [h]	X_{FF}[d] [%]	S_{FA}[e] [%]	TOF [h^{-1}]	Ref.
1	Pd/NPC	2-butanol	393	–	10	97	92	876	37
2	Co-Ru/C	Benzyl alcohol	423	–	12	98	>99.9	173.8	38
3	Au/TiO_2-R	Water	363	4[f]	4	>99.9	>99.9	91	39
4	15CMA	Formic acid	483	–	–	90	99	*ca.* 576	40
5	Ni-SAs/NC	Isopropanol	403	2	2	85.1	97.1	832	41
6[g]	Co-N-C-700	1,4-dioxane	423	0.5	6	>99.9	>99	88.2	42
7	(PhP)-Hf (1:1.5)	Isopropanol	393	–	2	99.2	98.4	16.8	43
8	ZrPN	Isopropanol	413	–	2	98	>99	22.5	44
9	M-ZrO_2-U-N	Isopropanol	383	–	6	92.4	97.1	15.1	45
10	M-MOF-808	Isopropanol	373	–	2	99.2	93.8	17.6	46
11	Al_2O_3-S(7)	Isopropanol	403	0.4	6	96.9	98.5	34	47
12	Fe-phen/C-800	Isopropanol	433	–	15	91.6	83	9.1	26
13	Fe-ZIF-8-800	Isopropanol	393	–	6	99.6	96.9	1882	This work
14[h]	Fe-ZIF-8-800		353		3	96.6	93.3	–	

[a]Reaction temperature. [b]Nitrogen pressure, "–" means not mentioned. [c]Reaction time. [d]FF conversion. [e]FA selectivity. [f]Carbon monoxide atmosphere. [g]Formic acid is used as hydrogen donor. [h]Fe-ZIF-8-800 has a higher iron loading of 0.54 mol%.

Table S16. CTH of FF to FA over Fe-based catalysts.

Entry	Catalyst	Solvent	T^a [K]	P^b [MPa]	t^c [h]	X_{FF}^d [%]	S_{FA}^e [%]	TOF [h^{-1}]	Ref.
1	Fe_3O_4@C	Isopropanol	473	2	4	93.6	98.9	0.77[f]	48
2	Fe_3O_4-12	Isopropanol	433	–	5	97.5	92.4	0.101[g]	49
3	Fe_3O_4/C	Isopropanol	473	2	4	76.4	98.5	0.46[h]	50
4	$LaFeO_3$	Isopropanol	453	1	3	90	94	1.73[i]	51
		Toluene		1[j]		20	18	0.074[k]	
5	$LaFeO_3$_N	Isopropanol	453	1	3	64.5	91.1	6.11[l]	52
6	$LaFeO_3$@C-M	Isopropanol	453	1	3	93.2	94.1	7.6[m]	53
7	HT_MgFe-3	Isopropanol	443	–[n]	6	97	92.8	0.01[o]	54
8	Fe-phen/C-800	Isopropanol	433	–	15	91.6	83.0	9.1[p]	26
9	Fe-ZIF-8-800	Isopropanol	393	–	6	99.6	96.9	1882[q]	This work

[a]Reaction temperature. [b]Nitrogen pressure, "–" means not mentioned. [c]Reaction time. [d]FF conversion. [e]FA selectivity. [f]TOF was calculated at 22.1% FF conversion and 200 °C for 1 h. [g]TOF was quoted in ref. [S6]. [h]TOF was calculated at 26% FF conversion and 200 °C for 1 h. [i]TOF was calculated at 9.6% FF conversion and 180 °C for 1 h. [j]Hydrogen atmosphere. [k]TOF was calculated at 20% FF conversion and 180 °C for 3 h. [l]TOF was calculated at 25.3% FF conversion and 180 °C for 1 h. [m]TOF was calculated at 22.7% FF conversion and 180 °C for 0.5 h. [n]Helium atmosphere. [o]TOF was

calculated at 20.2% FF conversion and 170 ºC for 0.5 h. [e]TOF was calculated at 25.6% FF conversion and 120 ºC for 0.5 h. [f]TOF was calculated at 20.5% FF conversion and 120 ºC for 5 min.

Table S17. Catalytic transfer hydrogenation of FF at various reaction conditions.[a]

Entry	T [ºC]	t [min]	Conv. [%]	Yield [%]	TOF [h^{-1}]
1	80	15	14.5	6.1	443.7
2	80	30	31.1	21.2	475.8
3	80	60	63.3	50.5	484.2
4	120	5	20.5	14.1	1881.9
5	120	10	33.3	23.3	1528.5
6	120	15	49.1	39.9	1502.5

[a]Reaction conditions: 0.5 mmol FF, 3 mL isopropanol solvent, 50 mg catalyst (0.13 mol% Fe).

Table S18. Bader charges of Fe and Zn sites for each state in Fe(II)-plN$_3$, Fe(II)-plN$_4$, Zn(II)-plN$_4$.

Fe(II)-plN$_3$	
**State**	**Bader Charge of Fe**
*(clean surface)	1.08
IPA*	1.20
TS1	1.22
depIPA_H*	1.18
depIPA_H_FF*	1.35
TS2	1.32
ACE_H_FFH*	1.31
H_FFH*	1.12
TS3	1.18
FA*	1.16
Fe(II)-plN$_4$	
**State**	**Bader Charge of Fe**
*(clean surface)	1.16
FF*	1.23
IPA_FF*	1.16
TS(P1)	1.27
ACE_FA*	1.24
FA*	1.23
IPA*	1.16
FF_IPA*	1.18
TS(P2)	1.25
FA_ACE*	1.25
ACE*	1.24
Zn(II)-plN$_4$	
**State**	**Bader Charge of Zn**
*(clean surface)	1.16
FF*	1.24
IPA_FF*	1.23
TS(P1)	1.24
ACE_FA*	1.23
FA*	1.24
IPA*	1.23
FF_IPA*	1.25
TS(P2)	1.25
FA_ACE*	1.24
ACE*	1.25

Table S19. Theoretical values of turnover frequencies (TOFs) on Fe(II)-plN_3, Fe(II)-plN_4, and Zn(II)-plN_4.

Models	TOF (s^{-1})
Fe(II)-plN_3	4.41E1
Fe(II)-plN_4	7.65E–5
Zn(II)-plN_4	1.15E–5

Table S20. The Fe and Zn contents in the reaction liquid after the first two reused cycle. The data were collected by ICP-AES analysis.

Runs	Fe Content [μg]	Zn Content [μg]	Percentage of Fe leaching [wt%]	Percentage of Zn leaching [wt%]
1	0.15	–	2.99×10^{-4}	–
2	0.05	0.25	1.02×10^{-4}	0.003

Table S21. Fe contents and textural properties of fresh, reused, and regenerated Fe-ZIF-8-800 catalysts.

Catalyst	Fe Content [wt%]	BET Surface Area [m^2/g]
fresh Fe-ZIF-8-800	0.073	403
used Fe-ZIF-8-800	0.045	33
regenerated Fe-ZIF-8-800[a]	0.091	178

[a]Re-pyrolysis of the used catalyst is proceeded by adding 0.033 wt% $Fe(NO_3)_3$ (the Fe dosage is only 0.01 wt%).

Table S22. Cutting energy and core-level width for XANES simulation of Fe and Zn reference compounds.

Reference Compounds	Cutting Energy (eV)	Core-level width (eV)
Fe foil	0	2
FeO	−4	2
Zn foil	−1	2
ZnO	1	1.5

Table S23. Microkinetic model parameters.

Parameter	Units	Values	Basis
Temperature	K	393	Experiments
Pressure	atm	4.2	Experiments
Feed composition	Molar ratio	IPA:FF=0.987:0.013	Experiments
Feed flow rate	cm^3/s	5E−2	Maintain conversion below 10%
Site density of each site type	mol/cm^2	7.55E−11	Model
Catalyst area/Volume	cm^{-1}	1.34E3	Experiments

Table S24. Elementary steps and corresponding kinetic parameters on Fe(II)-plN$_3$, Fe(II)-plN$_4$, and Zn(II)-plN$_4$ (T=393 K).

Elementary step	sticking coefficient/ pre-exponential factor	ΔH (eV)	ΔH$^{\neq}$ (eV)	ΔG (eV)	ΔG$^{\neq}$ (eV)
**Fe(II)-plN$_3$**					
$IPA_{(g)}$ + * = IPA*	5E−1	−0.87		−0.16	
IPA* = depIPA_H*	2.084E+10	0.05	0.23	0.07	0.28
depIPA_H* + $FF_{(g)}$ = depIPA_H_FF*	5E−1	−1.15		−0.35	
depIPA_H_FF* = ACE_H_FFH*	2.084E+10	0.18	0.78	0.08	0.87
ACE_H_FFH* = $ACE_{(g)}$ + H_FFH*	5E−1	0.67		0.07	
H_FFH* = FA*	2.084E+10	0.10	0.29	−0.01	0.32
FA* = $FA_{(g)}$ + *	5E−1	0.98		0.26	
IPA* + $FF_{(g)}$ = FF_IPA*	5E−1	−1.07		−0.23	
FF_IPA* = FA_ACE*	2.084E+10	0.19	1.20	0.06	1.25
FA_ACE* = ACE* + $FA_{(g)}$	5E−1	0.76		−0.05	
ACE* = ACE + *	5E−1	0.98		0.34	
$FF_{(g)}$ + * = FF*	5E−1	−1.00		−0.24	
FF* + $IPA_{(g)}$ = IPA_FF*	5E−1	−0.49		0.08	
IPA_FF* = ACE_FA*	2.084E+10	−0.28	0.90	−0.19	1.10
ACE_FA* = FA* + $ACE_{(g)}$	5E−1	0.78		0.04	
**Fe(II)-plN$_4$**					
$FF_{(g)}$ + * = FF*	5E−1	−0.69		0.00	
FF* + $IPA_{(g)}$ = IPA_FF*	5E−1	−0.37		0.31	
IPA_FF* = ACE_FA*	2.084E+10	−0.41	0.78	−0.39	0.89
ACE_FA* = FA* + $ACE_{(g)}$	5E−1	0.69		0.02	
FA* = $FA_{(g)}$ + *	5E−1	0.77		0.01	
$IPA_{(g)}$ + * = IPA*	5E−1	−0.66		0.01	
IPA* + $FF_{(g)}$ = FF_IPA*	5E−1	−0.96		−0.13	
FF_IPA* = FA_ACE*	2.084E+10	0.34	1.35	0.11	1.42
FA_ACE* = ACE* + $FA_{(g)}$	5E−1	0.67		−0.08	
ACE* = ACE + *	5E−1	0.60		0.03	
**Zn(II)-plN$_4$**					
$FF_{(g)}$ + * = FF*	5E−1	−0.68		0.04	
FF* + $IPA_{(g)}$ = IPA_FF*	5E−1	−0.42		0.18	
IPA_FF* = ACE_FA*	2.084E+10	−0.38	0.80	−0.36	0.97
ACE_FA* = FA* + $ACE_{(g)}$	5E−1	0.60		0.10	
FA* = $FA_{(g)}$ + *	5E−1	0.78		−0.02	
$IPA_{(g)}$ + * = IPA*	5E−1	−0.70		−0.05	

$IPA^* + FF_{(g)} = FF\_IPA^*$	5E−1	−0.94		−0.14	
$FF\_IPA^* = FA\_ACE^*$	2.084E+10	0.43	1.33	0.19	1.43
$FA\_ACE^* = ACE^* + FA_{(g)}$	5E−1	0.63		−0.06	
$ACE^* = ACE + *$	5E−1	0.58		0.02	

Table S25. Calculated relative solution-phase Gibbs free energies (in kcal/mol).

Molecule (M)	M	M···(^{i}PrOH)	M···(2 ^{i}PrOH)	M···(3 ^{i}PrOH)
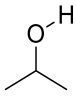 **a1**	0.0	4.6	11.7	N.A.
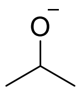 **b1**	0.0	–4.4	–4.2	–4.0
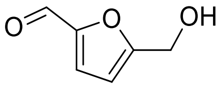 **c1**	0.0	4.6	10.6	N.A.
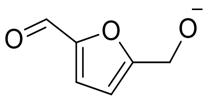 **d1**	0.0	–2.9	–2.8	0.4
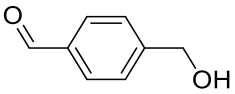 **e1**	0.0	5.2	11.6	N.A.
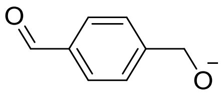 **f1**	0.0	–4.2	–3.1	–2.3
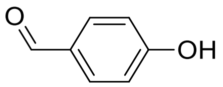 **g1**	0.0	6.8	13.8	N.A.
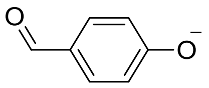 **h1**	0.0	2.8	5.6	9.2

N.A.=not applicable.

Table S26. Calculated thermodynamic corrections to Gibbs free energy (ΔG_{cor}), solution-phase single-point energies (ΔE_{sol}) and solution-phase Gibbs free energies (ΔG_{sol}) in Hartree.

Molecules	ΔG_{cor}	ΔE_{sol}	ΔG_{sol}
**a1**	0.081019	-194.3501563	-194.2661095
**a1**···(^{i}PrOH)	0.179184	-388.7071693	-388.5249575
**a1**···(2 ^{i}PrOH)	0.28218	-583.0649292	-582.7797213
**b1**	0.066502	-193.8330786	-193.7635488
**b1**···(^{i}PrOH)	0.166174	-388.2058761	-388.0366743
**b1**···(2 ^{i}PrOH)	0.267798	-582.5732182	-582.3023923
**b1**···(3 ^{i}PrOH)	0.369531	-776.9407746	-776.5682158
**c1**	0.080192	-457.8964114	-457.8131916
**c1**···(^{i}PrOH)	0.180312	-652.2552573	-652.0719175
**c1**···(2 ^{i}PrOH)	0.279983	-846.6114678	-846.328457
**d1**	0.066504	-457.393508	-457.3239761
**d1**···(^{i}PrOH)	0.166114	-651.7639102	-651.5947684
**d1**···(2 ^{i}PrOH)	0.267295	-846.1309433	-845.8606205
**d1**···(3 ^{i}PrOH)	0.37024	-1040.495012	-1040.121744
**e1**	0.109643	-460.1043024	-459.9916316
**e1**···(^{i}PrOH)	0.207342	-654.4598862	-654.2495164
**e1**···(2 ^{i}PrOH)	0.311539	-848.8199619	-848.5053951
**f1**	0.096306	-459.596199	-459.4968651
**f1**···(^{i}PrOH)	0.195003	-653.9676287	-653.7695979
**f1**···(2 ^{i}PrOH)	0.296759	-848.3337989	-848.034012
**f1**···(3 ^{i}PrOH)	0.397524	-1042.699486	-1042.298935
**g1**	0.082976	-420.8065389	-420.720535
**g1**···(^{i}PrOH)	0.182262	-615.1611636	-614.9758737
**g1**···(2 ^{i}PrOH)	0.281371	-809.5152167	-809.2308179
**h1**	0.070637	-420.3325661	-420.2589013
**h1**···(^{i}PrOH)	0.170578	-614.6941766	-614.5205707
**h1**···(2 ^{i}PrOH)	0.271065	-809.0563618	-808.7822689
**h1**···(3 ^{i}PrOH)	0.371499	-1003.417163	-1003.042636

Supplementary Note 1. Computational Details for pK_a prediction

The computational study for pK_a prediction was performed with Gaussian program [S7], M06-2X method [S8], SMD solvation model [S9] (solvent=2-propanol) for both geometry optimization and energy calculation. Geometry optimization and frequency analysis were conducted with def2-SVP basis set [S10] and solution-phase single-point energies were calculated with a larger basis set def2-TZVP [S10] based on the optimized structures (no imaginary frequency for each species). The thermodynamic correction to Gibbs free energy added by the solution-phase single-point energy and 1.9 kcal/mol (to account for the standard state change from 1 atm. to 1 M at 298.15 K) [S11] was used as the solution-phase Gibbs free energy of each optimized species. The pK_a values of HMF, 4-(hydroxymethyl)benzaldehyde and 4-hydroxybenzaldehyde in 2-propanol were calculated based on Eq. (1) by using the experimental pK_a value [S12] of 2-propanol in 2-propanol as a reference according to literature [S13]:

$$\mathrm{p}K_\mathrm{a}(\mathrm{HA}) = 22 + \frac{\Delta \mathrm{G}}{2.303 \times \mathrm{RT}} \qquad (1)$$

ΔG is the Gibbs free energy change of the deprotonation of concerned acid by 2-propanolate. However, the prediction based on the reaction formula HA + ^{i}PrO^{-} → A^{-} + ^{i}PrOH (HA is the concerned acid) has a large error probably because it omits the hydrogen bonding of solvent. For example, the experiment pK_a of 4-hydroxybenzaldehyde is 6.84 whereas the prediction based on the above reaction formula give a pK_a value of 3.18.

To solve this problem, we referred the cluster-continuum method [S13] and considered the hydrogen bonding of HA, $^{i}PrO^{-}$, $^{i}PrOH$ and A^{-} with several 2-propanol molecules to found the most stable forms for each of them. According to the number of lone pairs bear on hydroxyl/hydroxylate oxygen, hydrogen bonding with one to two 2-propanol molecules was considered for the neutral species, and hydrogen bonding with one to three 2-propanol molecules was considered for anionic species (**Table S25**). Thereafter, the ΔG^{*} is calculated based on the corrected reaction formulas (**Figure S28**) using the most stable forms to replace the ΔG. With the correction method, the pK_a value of 4-hydroxybenzaldehyde in 2-propanol is predicted to be 6.40, being much better.

References

[S1] James, J., Lin, Y. Kinetics of ZIF-8 thermal decomposition in inert, oxidizing, and reducing environments. *J. Phys. Chem. C* **120**, 14015–14026 (2016).

[S2] Muñoz-Gil, D., Figueiredo, F. High surface proton conduction in nanostructured ZIF-8. *Nanomaterials* **9**, 1369 (2019).

[S3] Chen, Y., Ji, S., Wang, Y., Dong, J., Chen, W., Li, Z., Shen, R., Zheng, L., Zhuang, Z., Wang, D., Li, Y. Isolated single iron atoms anchored on N-doped porous carbon as an efficient electrocatalyst for the oxygen reduction reaction. *Angew. Chem. Int. Ed.* **56**, 6937–6941 (2017).

[S4] Mironenko, A., Vlachos, D. Conjugation-driven "Reverse Mars-van Krevelen"-type radical mechanism for low-temperature C−O bond activation. *J. Am. Chem. Soc.* **138**, 8104−8113 (2016).

[S5] Panagiotopoulou, P., Martin, N., Vlachos, D. Effect of hydrogen donor on liquid phase catalytic transfer hydrogenation of furfural over a Ru/RuO_2/C catalyst. *J. Mol. Catal. A: Chem.* **392**, 223−228 (2014).

[S6] Ma, M., Hou, P., Zhang, P., Cao, J., Liu, H., Yue, H., Tian, G., Feng, S. Magnetic Fe_3O_4 nanoparticles as easily separable catalysts for efficient catalytic transfer hydrogenation of biomass-derived furfural to furfuryl alcohol. *Appl. Catal. A* **602**, 117709 (2020).

[S7] Frisch, M., Trucks, G., Schlegel, H., Scuseria, G., Robb, M., Cheeseman, J., Scalmani, G., Barone, V., Mennucci, B., Petersson, G., Nakatsuji, H., Caricato, M., Li, X., Hratchian, H., Izmaylov, A., Bloino, J., Zheng, G., Sonnenberg, J., Hada, M., Ehara, M., Toyota, K., Fukuda, R., Hasegawa, J., Ishida, M., Nakajima, T., Honda, Y., Kitao, O., Nakai, H., Vreven, T., Montgomery, J., Peralta, J., Ogliaro, F., Bearpark, M., Heyd, J., Brothers, E., Kudin, K., Staroverov, V., Keith, T., Kobayashi, R., Normand, J., Raghavachari, K., Rendell, A., Burant, J., Iyengar, S., Tomasi, J., Cossi, M., Rega, N., Millam, J., Klene, M., Knox, J., Cross, J., Bakken, V., Adamo, C., Jaramillo, J., Gomperts, R., Stratmann, R., Yazyev, O., Austin, A., Cammi, R., Pomelli, C., Ochterski, J., Martin, R., Morokuma, K., Zakrzewski, V., Voth, G., Salvador, P., Dannenberg, J., Dapprich, S., Daniels, A., Farkas, O., Foresman, J., Ortiz, J., Cioslowski, J., Fox, D. Gaussian 09, Revision D.01; Gaussian, Inc., Wallingford, CT (2013).

[S8] Zhao, Y., Truhlar, D. The M06 suite of density functionals for main group thermochemistry, thermochemical kinetics, noncovalent interactions, excited states, and transition elements: two new functionals and systematic testing of four M06-ClassFunctionals and 12 other functionals. *Theor. Chem. Acc.* **120**, 215–241 (2008).

[S9] Marenich, A., Cramer, C., Truhlar, D. Universal solvation model based on solute electron density and on a continuum model of the solvent defined by the bulk dielectric constant and atomic surface tensions. *J. Phys. Chem. B* **113**, 6378–6396 (2009).

[S10] Weigend, F., Ahlrichs, R. Balanced basis sets of split valence, triple zeta valence and quadruple zeta valence quality for H to Rn: design and assessment of accuracy. *Phys. Chem. Chem. Phys.* **7**, 3297–3305 (2005).

[S11] For recent examples considering energy correction for standard state change: (a) Li, H., Hall, M. Role of the chemically non-innocent ligand in the catalytic formation of hydrogen and carbon dioxide from methanol and water with the metal as the spectator. *J. Am. Chem. Soc.* **137**, 12330–12342 (2015). (b) Jiang, Y., Yan, L., Yu, H., Zhang, Q., Fu, Y. Mechanism of vanadium-catalyzed selective C−O and C−C cleavage of lignin model compound. *ACS Catal.* **6**, 4399–4410 (2016). (c) Yu, J., Zhang, S., Hong, X. Mechanisms and origins of chemo- and regioselectivities of Ru(II)-catalyzed decarboxylative C–H alkenylation of aryl carboxylic acids with alkynes: a computational study. *J. Am. Chem. Soc.* **139**, 7224–7243 (2017). (d) Jiang, Y., Li, G., Yang, D., Zhang, Z., Zhu, L., Fan, X., Bi, S. Mechanism of Cu-catalyzed aerobic C(CO)–CH_3 bond cleavage: a combined computational and experimental study. *ACS Catal.* **9**, 1066–1080 (2019).

[S12] Internet bond-energy databank (pK_a and BDE)--iBonD Home Page. http://ibond.chem.tsinghua.edu.cn or http://ibond.nankai.edu.cn.

[S13] Fu, Y., Liu, L., Li, R., Liu, R., Guo, Q. First-principle predictions of absolute pK_a's of organic acids in dimethyl sulfoxide solution. *J. Am. Chem. Soc.* **126**, 814–822 (2004).
