# Supplementary material for: Highly active, ultra-low loading single-atom iron catalysts for catalytic transfer hydrogenation
Source: Nat Commun. 2023 Oct 20;14:6666. doi: 10.1038/s41467-023-42337-9 (PMC10589291; doi:10.1038/s41467-023-42337-9)
Supplement: Supplementary file 3 — Description of Additional Supplementary Files [file 41467_2023_42337_MOESM3_ESM.pdf]

<u>Description of Additional Supplementary Files</u>

File Name: Supplementary Data 1

Description: The supplementary data file of "**Supplementary Data 1**" included the cartesian coordinates of all calculated species (In Angstrom) for pK_a prediction.
