# Supplementary material for: Highly active, ultra-low loading single-atom iron catalysts for catalytic transfer hydrogenation
Source: Nat Commun. 2023 Oct 20;14:6666. doi: 10.1038/s41467-023-42337-9 (PMC10589291; doi:10.1038/s41467-023-42337-9)
Supplement: Supplementary file 4 — Supplementary Data 1 [file 41467_2023_42337_MOESM4_ESM.pdf]

Supplementary Data 1. Cartesian Coordinates of All Calculated Species (In Angstrom)

a1

C	-0.00247900	0.04553900	0.36251800
H	0.00796200	0.08774300	1.46843000
O	0.10251500	1.35882900	-0.16547900
H	0.95629900	1.72144300	0.10524000
C	-1.34241300	-0.50341600	-0.08815700
H	-1.37574800	-0.55844100	-1.18739700
H	-1.51084300	-1.51031200	0.31846600
H	-2.15924500	0.15084900	0.24938200
C	1.15959700	-0.81805600	-0.10180500
H	2.11855700	-0.39148300	0.23108200
H	1.08378300	-1.83735800	0.30447700
H	1.17088600	-0.87746900	-1.20118400

a1···(^{i}PrOH)

C	-2.21519000	-0.21035900	-0.31804200
H	-2.48150600	-0.59468500	-1.31897600
O	-1.20736900	0.79180200	-0.45989100
H	-1.55223300	1.51168700	-1.00738100
C	-1.59860200	-1.33327600	0.49258400
H	-1.33377700	-0.97326600	1.49919200
H	-2.30433700	-2.16896000	0.59679800
H	-0.68741800	-1.70035900	-0.00134700
C	-3.45346000	0.37653900	0.33522600
H	-3.86217900	1.19994900	-0.27012400
H	-4.23716800	-0.38751000	0.44113600
H	-3.20565600	0.76733900	1.33397000
C	1.94939200	-0.09920400	0.21917700
H	1.40244100	-0.61480100	1.03392400
O	1.27268500	-0.30198900	-1.00794900
H	0.39397100	0.11615700	-0.92419000
C	3.32311700	-0.73011000	0.09092500
H	3.89184000	-0.23298800	-0.71061700
H	3.88800300	-0.63904500	1.02956000
H	3.23515600	-1.79726700	-0.16049300
C	2.02757700	1.38267200	0.55943700
H	1.01640100	1.80799600	0.64482800
H	2.55000000	1.54485300	1.51390400
H	2.56693400	1.92482900	-0.23330100

a1···(2 ^{i}PrOH)

C	0.17784200	2.52625500	0.05235200
H	-0.56281200	3.33126200	-0.09629600
O	-0.08622400	1.48973300	-0.90497800
H	0.04636300	1.84045500	-1.79924600
C	-0.02481400	1.90496500	1.41893000
H	0.69975400	1.08934400	1.56711400
H	0.12164100	2.65409800	2.20926300
H	-1.04432000	1.50084800	1.50365800
C	1.57618200	3.08195700	-0.14505100
H	1.69203700	3.49717200	-1.15795100
H	1.77371200	3.88788500	0.57624100
H	2.32343600	2.28703500	-0.00248400
C	-2.43604900	-0.73977300	0.09822500
H	-1.82344800	-0.69976400	1.02147400
O	-2.67285700	0.57753800	-0.36534000
H	-1.81214000	0.94104400	-0.64305600
C	-3.78021600	-1.35150000	0.44568300
H	-4.41352000	-1.40716300	-0.45359700
H	-3.65847000	-2.36559900	0.85219500
H	-4.29943500	-0.73640000	1.19516100
C	-1.68179800	-1.55326000	-0.94459300
H	-0.72062900	-1.07154000	-1.18488300
H	-1.47583000	-2.57260700	-0.58466000
H	-2.27391600	-1.62055800	-1.87124900
C	2.01510300	-1.61264000	-0.18282900
H	1.46406900	-2.11255700	-1.00289600
O	2.13476400	-0.22940000	-0.46451300
H	1.25358000	0.15973100	-0.60591400
C	3.42087600	-2.18196800	-0.13330900
H	3.99583300	-1.69040000	0.66709300
H	3.40173400	-3.26303100	0.06486100
H	3.94073300	-2.01063000	-1.08708900
C	1.26019600	-1.84615800	1.11734900
H	0.26506200	-1.37787600	1.07884300
H	1.12198600	-2.92109000	1.30843100
H	1.81518000	-1.40989500	1.96308700

b1

C	0.00001100	0.17022500	0.33186600
H	0.00004100	0.10396000	1.47389000
O	0.00007500	1.42188500	-0.14766700
C	-1.25456900	-0.63374700	-0.08638800
H	-1.28726800	-0.70194900	-1.18833000

H	-1.29457900	-1.65504200	0.33101200
H	-2.15861200	-0.09021200	0.23380100
C	1.25450000	-0.63385800	-0.08639500
H	2.15855300	-0.09004900	0.23329500
H	1.29473600	-1.65495000	0.33147600
H	1.28688800	-0.70255800	-1.18831100

b1···(^{i}PrOH)

C	1.82895600	0.09134200	0.17301900
H	1.34295300	0.53521600	1.08342500
O	1.02307400	0.19685700	-0.93936900
C	2.13030300	-1.37154700	0.52726500
H	2.63163700	-1.86212400	-0.32419700
H	2.77299700	-1.47117200	1.41734600
H	1.18867700	-1.90800100	0.71997600
C	3.13454500	0.86293900	-0.03607300
H	2.91708700	1.91913900	-0.25999400
H	3.79970500	0.82345100	0.84157100
H	3.67248900	0.44340900	-0.90289100
C	-2.04533400	0.14309800	-0.31508800
H	-2.18633300	0.54550800	-1.34633400
O	-1.07067600	-0.85254900	-0.30121800
H	-0.11256500	-0.39176300	-0.62721700
C	-3.37889300	-0.45162000	0.12092500
H	-3.29541000	-0.83808800	1.15002100
H	-4.19132600	0.29059500	0.09153500
H	-3.65134200	-1.29344700	-0.53392300
C	-1.65057300	1.32623200	0.56918000
H	-0.68636100	1.72760800	0.22461100
H	-2.40099800	2.13208500	0.54223400
H	-1.53440900	0.99045600	1.61316100

b1···(2 ^{i}PrOH)

C	0.42893000	2.34060000	-0.37410400
H	-0.09647800	2.99694100	-1.11092000
O	0.04941400	1.01813900	-0.54020300
C	0.03576200	2.84999900	1.01609700
H	0.59336000	2.29898200	1.79239900
H	0.23491800	3.92604100	1.14514600
H	-1.03795700	2.67037600	1.18109900
C	1.92689600	2.52726900	-0.61944500
H	2.19887900	2.15416000	-1.61910200
H	2.22596500	3.58563300	-0.55441500
H	2.51074800	1.95846000	0.12238600

C	-2.45337900	-0.60497900	0.21079500
H	-1.80769700	-0.64680900	1.11521000
O	-2.41605500	0.68233600	-0.34670500
H	-1.40630600	0.87283400	-0.48588300
C	-3.87988900	-0.91481200	0.63578400
H	-4.54548900	-0.88826100	-0.24221600
H	-3.95845900	-1.90845600	1.10141200
H	-4.23832000	-0.16331500	1.35535000
C	-1.92599300	-1.63881500	-0.78281400
H	-0.89401700	-1.39255200	-1.07779500
H	-1.93333300	-2.65633200	-0.36088900
H	-2.55159100	-1.63432400	-1.69061700
C	1.68657300	-1.60346400	0.23537100
H	0.66157300	-1.86405600	0.57714900
O	1.62506700	-0.88521900	-0.96962000
H	1.01984600	-0.06592800	-0.81463400
C	2.43431300	-2.90232700	-0.02194300
H	3.45615400	-2.68158900	-0.37036600
H	2.50107900	-3.51959900	0.88606600
H	1.92772400	-3.48650000	-0.80489200
C	2.34032300	-0.78227900	1.34280100
H	1.76583700	0.14228600	1.50113300
H	2.37511900	-1.33757200	2.29286600
H	3.36981800	-0.50961900	1.05848700

b1···(3 ^{i}PrOH)

C	-0.41097600	-0.20216100	2.38163700
H	0.27430000	0.53144300	2.86495300
O	-0.15160600	-0.25755100	1.00802400
C	-1.84169800	0.27030800	2.63903200
H	-2.55345800	-0.42974900	2.17103000
H	-2.06812600	0.33082000	3.71505100
H	-1.99551500	1.26577600	2.19581300
C	-0.15489500	-1.56301400	3.02762400
H	0.87323800	-1.89652300	2.81912500
H	-0.29629800	-1.53539400	4.11946900
H	-0.84666800	-2.31251500	2.60771700
C	0.46837600	2.64619400	-0.52934500
H	0.69184900	2.11023900	-1.47718700
O	-0.67092500	2.08920700	0.08066700
H	-0.44696600	1.15655300	0.40971800
C	0.15642400	4.09279100	-0.87549800
H	-0.04619000	4.66361700	0.04491300
H	0.99466800	4.56928900	-1.40458400

H	-0.73705100	4.14877400	-1.51524800
C	1.69155200	2.52642200	0.37305500
H	1.90742400	1.47027500	0.59399600
H	2.58228000	2.96773600	-0.10062400
H	1.50821200	3.05120000	1.32510900
C	2.36483400	-1.18937800	-0.76522300
H	1.96352200	-0.32079200	-1.33197300
O	2.27586900	-0.93721100	0.61707700
H	1.30639800	-0.68292600	0.79985400
C	3.83142200	-1.35090700	-1.12837500
H	4.26374800	-2.19351800	-0.56529100
H	3.95996500	-1.54832000	-2.20276400
H	4.39695300	-0.44260700	-0.87140600
C	1.54707800	-2.41951000	-1.14651500
H	0.49091200	-2.28598800	-0.86670700
H	1.59464800	-2.61771000	-2.22872300
H	1.93487400	-3.30305700	-0.61358300
C	-2.36588300	-0.63779200	-1.20294200
H	-2.64133500	0.29002900	-0.66077800
C	-3.60556700	-1.17571400	-1.89631000
H	-4.01277500	-0.44641100	-2.61198600
H	-3.36000900	-2.09998000	-2.44362200
H	-4.38634700	-1.41325300	-1.15860500
C	-1.26854800	-0.27235000	-2.19731300
H	-1.58318500	0.54852100	-2.86091400
H	-0.36640600	0.05135400	-1.65623600
H	-1.00776800	-1.14382600	-2.82016200
O	-1.91259000	-1.60544900	-0.28305400
H	-1.19358600	-1.14436100	0.26297400

c1

C	-0.98276800	-0.08862600	0.02587200
C	-0.64250300	1.23929800	0.03005000
C	0.78005800	1.27520500	0.01650900
C	1.19565800	-0.03088900	0.00523700
O	0.11366500	-0.85838400	0.01074300
H	-1.33601000	2.07519300	0.04020700
H	1.43002200	2.14745200	0.01429400
C	2.51565600	-0.64065000	-0.01729100
H	2.51485300	-1.75303200	-0.02615800
O	3.54526600	-0.00027300	-0.02651000
C	-2.30396800	-0.77766700	0.04219100
H	-2.32498700	-1.51740700	-0.77921100
H	-2.39744100	-1.34313000	0.98764900

O	-3.29665700	0.20318700	-0.09053000
H	-4.15742600	-0.22535100	-0.00182000

c1···(^{i}PrOH)

C	0.53628300	1.21464800	-0.02519400
C	0.65311200	1.10288800	1.33505000
C	1.74375900	0.21552900	1.55500800
C	2.20775600	-0.13804700	0.31413000
O	1.46577800	0.47478100	-0.64659300
H	0.02249100	1.58557900	2.07601800
H	2.14494700	-0.12735900	2.50642100
C	3.29374700	-1.01295700	-0.09427000
H	3.84027200	-1.47809500	0.75425100
O	3.59441500	-1.23123500	-1.24832400
C	-0.42310300	1.96619200	-0.88322700
H	0.04907900	2.89874400	-1.23900200
H	-0.65949400	1.35337400	-1.77091700
O	-1.56307000	2.24003400	-0.10239300
H	-2.16381100	2.80328500	-0.60997800
H	-2.15865700	0.67814800	0.76444400
O	-2.28723400	-0.23959700	1.06027100
C	-1.98645100	-1.07674500	-0.04150800
H	-0.93770600	-0.90950900	-0.36272800
C	-2.11193500	-2.51283300	0.43048800
C	-2.90497700	-0.77023800	-1.21610000
H	-3.14155400	-2.70969500	0.76799600
H	-1.86744600	-3.21634000	-0.37785200
H	-1.43038100	-2.69924600	1.27344200
H	-2.82426700	0.29151300	-1.49682100
H	-2.64981500	-1.38027500	-2.09514800
H	-3.95191200	-0.97261500	-0.94007100

c1···(2 ^{i}PrOH)

C	-1.79745400	-0.44837100	0.40714600
C	-2.29294300	0.18027300	1.51931100
C	-3.70503700	0.22368800	1.33894600
C	-3.95658600	-0.37949900	0.13470100
O	-2.78815900	-0.79002400	-0.42841300
H	-1.71376200	0.56510800	2.35446800
H	-4.45097900	0.64753100	2.00793800
C	-5.19954900	-0.62009300	-0.57995100
H	-6.10357800	-0.25497300	-0.04661200
O	-5.26735800	-1.16599400	-1.66003600
C	-0.40514400	-0.78722700	-0.01343700

H	-0.34364800	-1.85635500	-0.27601600
H	-0.14239200	-0.21255600	-0.91550900
O	0.52259700	-0.44059700	0.99069600
H	0.36019300	1.47496300	0.93155000
O	0.29218500	2.27132700	0.38216800
C	1.47234000	2.32756800	-0.40453900
H	1.53682100	1.42328600	-1.04119000
C	1.35795300	3.54383600	-1.30304500
C	2.71061800	2.37581300	0.47858800
H	1.28815900	4.45877200	-0.69388800
H	2.23337100	3.62853000	-1.96225600
H	0.45547300	3.47663500	-1.92782400
H	2.74852900	1.49804600	1.14247800
H	3.62962500	2.37919900	-0.12605100
H	2.69556300	3.28233600	1.10416800
H	0.40712100	-1.02280400	1.75779400
H	2.14247900	-0.51168500	0.04797800
O	2.82714500	-0.71860800	-0.60989500
C	3.45068100	-1.92752300	-0.20985400
H	2.71240400	-2.75324300	-0.23969600
C	4.54801700	-2.22445300	-1.21367600
C	3.98577900	-1.80856600	1.20942600
H	5.30089100	-1.42101100	-1.19595800
H	5.04642700	-3.17648000	-0.98293900
H	4.13306600	-2.28620900	-2.23022500
H	3.16981600	-1.57485800	1.91130000
H	4.45935800	-2.74555300	1.53781300
H	4.73173000	-1.00018900	1.26482300

d1

C	-0.96176600	-0.07139600	0.00002400
C	-0.83965500	1.29773400	-0.00005800
C	0.55249000	1.57343400	0.00006100
C	1.18630300	0.35357100	-0.00003800
O	0.25666000	-0.64427400	0.00003000
H	-1.67408900	1.99189900	-0.00011200
H	1.04763900	2.54285000	0.00008700
C	2.59146300	0.01602100	-0.00010400
H	3.26275700	0.90338500	0.00001000
O	3.03486200	-1.11639600	0.00009500
C	-2.21848300	-0.91795200	0.00000400
H	-2.05968500	-1.62403600	-0.87703400
H	-2.05974500	-1.62401300	0.87706900
O	-3.33889600	-0.20165000	-0.00004400

d1···(^{i}PrOH)

C	1.10149500	-0.47806700	0.26844500
C	1.51678500	-1.51172300	-0.53519200
C	2.87193700	-1.23677700	-0.86194400
C	3.18731200	-0.05663500	-0.23386900
O	2.10299300	0.39944000	0.45386400
H	0.90119700	-2.35297200	-0.83836900
H	3.54672400	-1.82485100	-1.48125900
C	4.41600800	0.70745600	-0.19828500
H	5.24257600	0.25355300	-0.78797400
O	4.56401700	1.74943900	0.40885200
C	-0.22796700	-0.22877200	0.92759800
H	-0.00958900	-0.13420800	2.02122000
H	-0.52338800	0.80872500	0.62292000
O	-1.14671900	-1.18191600	0.62016400
H	-2.01738900	-0.53947200	-0.28457900
O	-2.66929000	0.05168400	-0.87209300
C	-3.64252300	0.54450500	0.00488400
H	-3.16477500	1.11041300	0.83650600
C	-4.54225900	1.50908100	-0.75357200
C	-4.44304300	-0.59276700	0.63611500
H	-5.04653100	0.97914500	-1.57794900
H	-5.31050100	1.95028800	-0.10091700
H	-3.94563300	2.32415800	-1.19023900
H	-3.75373500	-1.27086500	1.16011000
H	-5.18950600	-0.22408300	1.35676900
H	-4.96392900	-1.16681100	-0.14762200

d1···(2 ^{i}PrOH)

C	1.39496500	-0.74192500	0.52001000
C	1.90899100	-0.13728600	1.64094200
C	3.31428300	-0.06812200	1.43909700
C	3.55505400	-0.63394000	0.21157400
O	2.37995400	-1.04263100	-0.34266200
H	1.32912300	0.20883900	2.49126600
H	4.06882400	0.34605500	2.10507400
C	4.78667200	-0.83210000	-0.52531400
H	5.69084500	-0.46190700	0.00578100
O	4.85718400	-1.35097800	-1.62096600
C	-0.01923700	-1.06495000	0.14171900
H	-0.12707200	-0.78089800	-0.93266500
H	-0.12058800	-2.17468700	0.14774600
O	-0.91680500	-0.44159100	0.97208700

H	-2.15161700	-1.31180300	0.89786200
O	-2.95789600	-1.88979200	0.65790400
C	-3.65357000	-1.17194900	-0.33116400
H	-2.93544600	-0.74709100	-1.06398300
C	-4.57393700	-2.13241000	-1.06715300
C	-4.42529500	-0.00591100	0.28033300
H	-5.30468700	-2.56431000	-0.36439200
H	-5.12501700	-1.62780500	-1.87442600
H	-3.99291800	-2.95828000	-1.50370800
H	-3.73217400	0.68869600	0.77784600
H	-4.97927100	0.56014500	-0.48469400
H	-5.14281200	-0.38059900	1.02820300
H	-1.28754900	0.78411300	0.21273100
O	-1.46808700	1.62926300	-0.34336400
C	-0.44591200	2.54760100	-0.04378100
H	-0.05624500	2.34779800	0.97568500
C	0.71659700	2.41383000	-1.02455700
C	-1.02286400	3.95605200	-0.06402200
H	0.36369900	2.60990700	-2.05014300
H	1.52714400	3.12248600	-0.79380900
H	1.13753300	1.39658500	-1.00052400
H	-1.84380500	4.04424300	0.66278500
H	-0.25995500	4.71100600	0.17758600
H	-1.42729300	4.18000400	-1.06430200

d1···(3 ^{i}PrOH)

C	-1.76095500	0.38987100	-0.76826800
C	-1.93044900	1.13663900	0.37327600
C	-3.32903600	1.14954700	0.62780700
C	-3.91085200	0.41496900	-0.37518900
O	-2.95006900	-0.04310100	-1.22251700
H	-1.13093800	1.59273900	0.95240400
H	-3.85565600	1.63487500	1.44720400
C	-5.29592200	0.07718800	-0.63785900
H	-6.00887900	0.49094500	0.10839200
O	-5.67235000	-0.59392400	-1.57695300
C	-0.53737600	-0.01162400	-1.54067600
H	-0.74805700	-1.02149400	-1.95539900
H	-0.47536300	0.65811100	-2.42683700
O	0.60878700	0.02319000	-0.77513700
H	1.99355000	-0.20673400	-1.54598200
O	2.89556600	-0.43796700	-1.91774400
C	3.44362000	-1.40673300	-1.04902500
H	2.73129100	-2.24755200	-0.93077900

C	4.71394400	-1.94344000	-1.68534200
C	3.70737400	-0.82548600	0.33619000
H	5.44117800	-1.12673400	-1.81944000
H	5.17590600	-2.72156400	-1.06028900
H	4.49655600	-2.37338000	-2.67438500
H	2.79990100	-0.35011100	0.73903900
H	4.02705700	-1.60842300	1.04139700
H	4.49976300	-0.06077700	0.28472600
H	0.62216900	-1.31602700	0.03135000
O	0.57622100	-2.14832400	0.59852100
C	-0.23070100	-1.84508300	1.71428200
H	-0.23710200	-0.74783400	1.87218400
C	-1.66600100	-2.30652100	1.47861100
C	0.36793100	-2.49877700	2.95108500
H	-1.69180400	-3.40264800	1.36583000
H	-2.32765600	-2.02761100	2.31348200
H	-2.07095000	-1.86430800	0.55488000
H	1.39042700	-2.13020800	3.12134900
H	-0.23290000	-2.29071100	3.84891000
H	0.41647500	-3.59080000	2.81203700
H	0.90442100	1.02167400	0.41514100
O	1.14827200	1.69312800	1.12172200
C	1.50459400	2.87359700	0.43259000
H	0.74988000	3.07982300	-0.35464300
C	1.49184800	4.02573300	1.42255500
C	2.86195400	2.71916500	-0.24298100
H	2.20174800	3.82214200	2.24008700
H	1.78030500	4.97060400	0.93952600
H	0.49142400	4.15212200	1.86288000
H	2.86156700	1.84452400	-0.91084800
H	3.11828600	3.61134500	-0.83483700
H	3.64615800	2.56973800	0.51712900

e1

C	0.81136700	-1.03342400	0.00016600
C	-0.55994200	-1.28859500	0.00002600
C	-1.47158300	-0.23092000	-0.00005100
C	-1.00546200	1.09291000	-0.00007800
C	0.35749300	1.34429500	-0.00006700
C	1.27837800	0.28254100	0.00008500
H	1.52771000	-1.85433400	0.00028100
H	-0.92794500	-2.31791600	0.00002400
H	-1.72893000	1.91056800	-0.00018700
H	0.72475700	2.37368300	-0.00010800

C	-2.92233300	-0.51307500	-0.00002600
H	-3.19392300	-1.59349600	0.00000600
C	2.75264300	0.59109300	0.00023500
H	2.97737400	1.21346200	-0.88566800
H	2.97748900	1.21238000	0.88685300
O	3.50085400	-0.59675600	-0.00039900
H	4.43699800	-0.36053700	0.00016600
O	-3.78046600	0.34016000	0.00001000

e1···(^{i}PrOH)

C	0.81364000	0.09355800	-0.73287900
C	1.92576600	-0.73343600	-0.61143900
C	3.03341300	-0.31574800	0.13406400
C	3.01855500	0.93543700	0.76250900
C	1.90594500	1.75950800	0.64311300
C	0.79586900	1.34622800	-0.10569100
H	-0.05486500	-0.23680900	-1.30263900
H	1.94747600	-1.71272300	-1.09370000
H	3.88476900	1.25656400	1.34643800
H	1.89193000	2.73502400	1.13485800
C	4.22067800	-1.18739000	0.26670600
H	5.04675400	-0.76941900	0.88628700
C	-0.38205000	2.27636000	-0.23668100
H	-0.67521000	2.63206000	0.76620300
H	-0.06897700	3.16237100	-0.81633300
O	-1.45218500	1.61795400	-0.87735800
H	-2.17255800	2.24879800	-1.01185700
O	4.32350900	-2.27868600	-0.24540100
H	-1.94499200	0.08182500	0.00081200
O	-2.29922100	-0.77566900	0.29681400
C	-3.65430600	-0.81687100	-0.11274400
H	-3.71573500	-0.67070700	-1.20921100
C	-4.19037700	-2.19627200	0.21959800
C	-4.45313500	0.28763900	0.56594900
H	-4.13814900	-2.36839300	1.30623000
H	-5.23657200	-2.30196500	-0.10049000
H	-3.59204800	-2.97197200	-0.28017400
H	-4.02238600	1.27329200	0.33083100
H	-5.50267700	0.28497300	0.23640200
H	-4.42756800	0.15420500	1.65886800

e1···(2 ^{i}PrOH)

C	0.49059300	-0.95859200	0.87042200
C	1.47829800	-1.52579700	0.07621400

C	2.77800100	-1.00065300	0.09628800
C	3.07957900	0.09207200	0.91308400
C	2.08357400	0.66744400	1.70007000
C	0.78639700	0.14740800	1.68354700
H	-0.52161900	-1.36951600	0.86699800
H	1.25980600	-2.38233500	-0.56470700
H	4.09486600	0.49655900	0.92149100
H	2.31133000	1.53390100	2.32477900
C	3.84019600	-1.59488600	-0.74568500
H	4.83921400	-1.11079700	-0.65506000
C	-0.29640000	0.77173300	2.52304000
H	-0.50798100	0.13343500	3.39840200
H	0.03727000	1.75291700	2.89291100
O	-1.47073000	0.90941400	1.73111000
H	-2.11297200	1.45652500	2.20810800
O	3.67207100	-2.53568600	-1.48649100
H	-2.45950700	-0.63910200	1.35160200
O	-2.88561800	-1.42084400	0.95902800
C	-3.04680300	-1.15290800	-0.42712400
H	-2.05847800	-0.93851200	-0.87509300
C	-3.61694500	-2.40467800	-1.06552800
C	-3.93605800	0.06289000	-0.64363500
H	-4.59512000	-2.64397200	-0.61941600
H	-3.75088500	-2.26720000	-2.14775000
H	-2.94628400	-3.26129900	-0.90416200
H	-3.50851300	0.94951300	-0.15063600
H	-4.03762900	0.29121700	-1.71503200
H	-4.93894300	-0.11781500	-0.22518000
H	-1.13503200	1.20013000	-0.12153100
O	-0.98352300	1.33943200	-1.07274100
C	0.30421300	1.90563800	-1.24809500
H	1.07705100	1.16957800	-0.95166300
C	0.46796900	2.20341900	-2.72640100
C	0.46611200	3.14949500	-0.38653300
H	-0.29059500	2.93232200	-3.05213700
H	1.46465000	2.61747500	-2.93505700
H	0.34146800	1.28568200	-3.31886600
H	0.28489900	2.91111300	0.67365500
H	1.48115100	3.56502900	-0.47419600
H	-0.25809300	3.92112300	-0.69269000

f1

C	-0.85669800	-1.03399700	0.00020800
C	0.51325900	-1.28899300	0.00025100

C	1.42296800	-0.22605300	0.00004300
C	0.94729400	1.09792700	-0.00000900
C	-0.41719600	1.34120800	-0.00002600
C	-1.33731200	0.27665400	0.00001500
H	-1.60332600	-1.82959200	0.00012000
H	0.88955200	-2.31627100	0.00011500
H	1.66883000	1.91803400	-0.00004600
H	-0.78966800	2.37065800	0.00020600
C	2.86826800	-0.50470600	-0.00010100
H	3.13795700	-1.58646800	-0.00057500
C	-2.85687100	0.50300300	0.00010400
H	-3.00555700	1.21289500	0.87755400
H	-3.00512100	1.21351200	-0.87669100
O	-3.60922100	-0.59004700	-0.00037600
O	3.73485400	0.34341900	-0.00007300

f1···(^{i}PrOH)

C	-0.98168300	0.21884500	-1.01526900
C	-2.22090600	-0.40525900	-0.92392800
C	-3.04257400	-0.16922200	0.18619600
C	-2.60960700	0.69775800	1.19977100
C	-1.37027900	1.31860500	1.10120900
C	-0.54116700	1.08521400	-0.00630900
H	-0.31781500	0.05328600	-1.86493500
H	-2.57133500	-1.08270800	-1.70611900
H	-3.25409800	0.87724100	2.06477500
H	-1.03171400	1.99388000	1.89233300
C	-4.35701300	-0.82650000	0.30048700
H	-4.93022400	-0.57717600	1.22350500
C	0.81213700	1.77397000	-0.13574600
H	0.57406200	2.87075200	-0.14937400
H	1.31513400	1.63281900	0.85649100
O	1.57271500	1.36566300	-1.18329400
O	-4.81810900	-1.58706400	-0.52238800
H	2.76220900	0.93583400	-0.57943200
O	3.61183600	0.57901100	-0.05043700
C	3.58996600	-0.81673600	-0.13957600
H	3.54124000	-1.13812100	-1.20443000
C	4.88285400	-1.36398700	0.44741700
C	2.36719300	-1.40121800	0.56505300
H	4.96204200	-1.07282600	1.50742000
H	4.92998400	-2.46149600	0.38331400
H	5.75070400	-0.94537200	-0.08413200
H	1.45225700	-0.97960800	0.12251700

H	2.32491500	-2.49795900	0.47542900
H	2.38757300	-1.13824400	1.63576200

f1···(2 ^{i}PrOH)

C	0.74925300	-0.72142200	-0.45560200
C	1.92367800	-1.39403800	-0.77342300
C	2.88153800	-1.64239000	0.21851200
C	2.65049100	-1.21032300	1.53104700
C	1.47416700	-0.53837700	1.84320100
C	0.50982600	-0.28827100	0.85617600
H	-0.00071900	-0.51563500	-1.22028100
H	2.11701800	-1.73490800	-1.79284600
H	3.40221200	-1.40237700	2.30145500
H	1.29487100	-0.19708600	2.86655000
C	4.13220100	-2.35391300	-0.10444600
H	4.82483500	-2.50117900	0.75637100
C	-0.78620000	0.42853000	1.21116400
H	-1.36190600	-0.28835700	1.84569300
H	-0.50998300	1.25222100	1.91054000
O	-1.51947700	0.86642500	0.13840200
O	4.42393600	-2.76536300	-1.20577800
H	-2.19253800	-0.38272500	-0.36912800
O	-2.63057400	-1.27447800	-0.62173400
C	-4.02114200	-1.11724800	-0.49680700
H	-4.38808800	-0.35176100	-1.21432100
C	-4.68561300	-2.43865000	-0.84682700
C	-4.39468900	-0.65031100	0.90674600
H	-4.35638100	-3.22002000	-0.14329700
H	-5.78223500	-2.36529900	-0.80040300
H	-4.40038100	-2.75319000	-1.86194300
H	-3.87423000	0.29188500	1.13372100
H	-5.47827000	-0.48406100	1.00641600
H	-4.08765700	-1.40550900	1.64858000
H	-0.59711300	1.72924100	-0.73688400
O	0.07807300	2.30774500	-1.23565700
C	0.74396300	3.07524500	-0.26507600
H	1.24401700	2.40932300	0.47234000
C	1.82167700	3.89361100	-0.95666700
C	-0.24053200	3.95886200	0.49524000
H	1.36260400	4.56383100	-1.70115800
H	2.38756600	4.50524600	-0.23855900
H	2.52618700	3.23218400	-1.48257900
H	-1.04251300	3.33788300	0.92239500
H	0.24952600	4.51416300	1.30960900

H	-0.70420000	4.68367900	-0.19355400

f1···(3 ^{i}PrOH)

C	-2.13022800	-0.01271300	-0.47738700
C	-3.51290100	-0.07521600	-0.61383400
C	-4.32829700	-0.15094900	0.52199000
C	-3.74488000	-0.16379200	1.79604700
C	-2.36306300	-0.10108000	1.92677800
C	-1.54039100	-0.02414400	0.79358600
H	-1.48865100	0.04734700	-1.35759100
H	-3.97982200	-0.06628000	-1.60100000
H	-4.38430400	-0.22371900	2.68078600
H	-1.90690500	-0.11106100	2.92036300
C	-5.79649800	-0.21804800	0.39102300
H	-6.35265100	-0.27689900	1.35515700
C	-0.03156900	0.05401100	0.96078700
H	0.16489300	0.95933800	1.57904800
H	0.26026600	-0.80142700	1.61078700
O	0.66870000	0.07387900	-0.22634100
O	-6.39228000	-0.21310800	-0.66338500
H	0.29755400	1.45890000	-0.89427600
O	0.05620300	2.37097000	-1.24228300
C	1.18454200	3.20012000	-1.08634300
H	2.06409100	2.74508500	-1.58792300
C	0.88714400	4.52883100	-1.76045900
C	1.53482300	3.37527100	0.38715400
H	0.02310700	5.01036300	-1.27495000
H	1.74618200	5.21272900	-1.70051200
H	0.63901900	4.37193200	-2.82064800
H	1.78438400	2.40097600	0.83255900
H	2.40275700	4.03973100	0.51959200
H	0.67689100	3.80477700	0.92929200
H	0.35989600	-1.34141900	-0.86409100
O	0.14215400	-2.28902200	-1.12109800
C	1.23622600	-3.08538600	-0.73194800
H	1.43763600	-2.94907600	0.35215200
C	0.86585600	-4.54096200	-0.96170300
C	2.49812100	-2.69597800	-1.49602100
H	0.66251100	-4.70965300	-2.03141500
H	1.67612200	-5.21636100	-0.65060900
H	-0.04201800	-4.80124700	-0.39766100
H	2.73879900	-1.63569700	-1.32417300
H	3.36240400	-3.30262200	-1.18300100
H	2.34081700	-2.84448700	-2.57661900

H	2.21484800	0.31533900	-0.11900100
O	3.21820400	0.41565000	-0.07519400
C	3.66925600	-0.34861500	1.01944500
H	3.11573300	-1.31048100	1.05322700
C	5.14362000	-0.65531400	0.81266100
C	3.42676100	0.38252400	2.33589700
H	5.71135100	0.28536300	0.73187300
H	5.55477000	-1.23898000	1.64922000
H	5.29346000	-1.22439600	-0.11698500
H	2.35637100	0.60455900	2.46541600
H	3.75730100	-0.21749700	3.19758200
H	3.97819800	1.33654000	2.34376700

g1

C	-1.21529500	1.18506500	-0.00006000
C	0.16557900	1.33744600	-0.00004500
C	1.00476300	0.21725200	-0.00002700
C	0.44098800	-1.06904200	-0.00007300
C	-0.93336100	-1.23302500	-0.00006900
C	-1.76944300	-0.10249300	0.00001600
H	-1.87731600	2.05385800	-0.00010200
H	0.60223400	2.33952500	-0.00005800
H	1.10320400	-1.93727100	-0.00011500
H	-1.39042300	-2.22368000	-0.00008500
C	2.46581000	0.39534000	0.00004600
H	2.81027200	1.45511200	-0.00006600
O	3.26709900	-0.51370400	0.00009100
O	-3.09599500	-0.31176900	0.00005500
H	-3.57105400	0.53297700	0.00053600

g1···(^{i}PrOH)

C	-0.35101800	-0.29975100	-0.73857900
C	-1.54071700	-0.99750000	-0.58176200
C	-2.66630600	-0.37299500	-0.02873000
C	-2.58776100	0.96934400	0.36845200
C	-1.40573700	1.67834200	0.21731200
C	-0.28530700	1.04179500	-0.33812200
H	0.53961200	-0.76495300	-1.16434400
H	-1.60226900	-2.04411800	-0.89098000
H	-3.46954900	1.44886800	0.79813500
H	-1.33327500	2.72443700	0.52299200
C	-3.92155100	-1.12782100	0.13059200
H	-3.88222000	-2.18515700	-0.21849200
O	-4.94015600	-0.67040400	0.60033700

O	0.88838700	1.68515300	-0.51132300
H	0.83954100	2.60080000	-0.19579100
H	2.40012900	0.58074600	-1.12271200
O	2.92625800	-0.23091200	-1.06002800
C	3.13463100	-0.47664900	0.32264100
H	2.15387900	-0.54712700	0.83375700
C	3.84036300	-1.81383700	0.44046500
C	3.93158600	0.65526300	0.95383800
H	4.80866300	-1.77481200	-0.08260500
H	4.02252200	-2.06999400	1.49365200
H	3.23579400	-2.61270500	-0.01306900
H	3.39506700	1.61033700	0.84575300
H	4.09644300	0.47591500	2.02642300
H	4.91065500	0.74992000	0.45875800

g1···(2 ^{i}PrOH)

C	-0.47961500	-0.79089400	-0.40022800
C	-1.64419600	-1.08681200	-1.09607300
C	-2.87506700	-1.15079600	-0.43017700
C	-2.93093000	-0.91955100	0.95133300
C	-1.77520400	-0.62159300	1.65772000
C	-0.55388400	-0.55218400	0.97573700
H	0.49055800	-0.74291400	-0.89579000
H	-1.60254400	-1.27476400	-2.17187900
H	-3.89426800	-0.97451300	1.46205600
H	-1.80164800	-0.43061700	2.73287900
C	-4.10266100	-1.46596400	-1.18409200
H	-3.95523800	-1.63869700	-2.27473000
O	-5.20565700	-1.54092400	-0.68993000
O	0.60006900	-0.22800400	1.61238800
H	0.46823000	-0.16164400	2.57222000
H	2.11668800	-1.17884000	0.79803300
O	2.67463500	-1.79207600	0.29642300
C	3.93585600	-1.18052200	0.07530000
H	4.33674200	-0.80483100	1.03593600
C	4.86860300	-2.25744800	-0.44626600
C	3.81544000	-0.01154200	-0.89152600
H	4.48317100	-2.66104300	-1.39570800
H	5.87583200	-1.85424800	-0.62242400
H	4.94386900	-3.08448800	0.27448300
H	3.10257000	0.74021700	-0.52093800
H	4.78780100	0.48296500	-1.03609000
H	3.45876900	-0.36764700	-1.87139700
H	1.05666700	1.47837300	0.66276100

O	1.05138400	2.03801200	-0.12826100
C	0.29875400	3.21049300	0.14162800
H	0.84424400	3.84010500	0.87067500
C	0.18234200	3.97315400	-1.16369800
C	-1.05939500	2.85852500	0.72849300
H	-0.36341700	3.36853900	-1.90468600
H	-0.35550900	4.92052900	-1.01851700
H	1.17996100	4.19753200	-1.56833700
H	-0.94266200	2.30029900	1.67093500
H	-1.64213700	3.76645200	0.94348700
H	-1.63139600	2.23397400	0.02315100

h1

C	1.25867500	1.19914700	0.00013600
C	-0.11022800	1.33864600	0.00019900
C	-0.96899500	0.21448500	0.00001000
C	-0.37705000	-1.07353100	0.00038500
C	0.98674100	-1.23278800	0.00019100
C	1.89882500	-0.10180400	-0.00043200
H	1.91320600	2.07480400	0.00034700
H	-0.55592800	2.33939700	0.00031000
H	-1.03696900	-1.94603800	0.00058400
H	1.43654700	-2.22944800	0.00032100
C	-2.40213500	0.38429200	-0.00010100
H	-2.74033400	1.44977800	0.00006900
O	-3.23525000	-0.51305400	-0.00028600
O	3.14381000	-0.24434300	-0.00020700

h1···(^{i}PrOH)

C	-0.38683600	-0.47687800	-0.64084700
C	-1.60628800	-1.08880000	-0.43806800
C	-2.72885500	-0.36200800	0.01003800
C	-2.57716700	1.02261800	0.25093200
C	-1.36793800	1.64972100	0.05522100
C	-0.20609600	0.93109700	-0.40373900
H	0.47808400	-1.04358700	-0.99286900
H	-1.71388800	-2.16165500	-0.62892400
H	-3.44611600	1.58840500	0.59802600
H	-1.25088200	2.72014400	0.24145700
C	-4.00127300	-1.02728300	0.21248400
H	-3.99304000	-2.12153400	-0.01202600
O	-5.03244000	-0.49563600	0.59377300
O	0.90750300	1.51882700	-0.58804700
H	2.11869200	0.50243600	-0.94215300

O	2.84809500	-0.17864000	-0.99084000
C	3.23060600	-0.42622400	0.34437500
H	2.33975100	-0.71392700	0.94128800
C	4.20261700	-1.59291000	0.34705800
C	3.83580000	0.82271600	0.97463900
H	5.09347100	-1.34003800	-0.24971300
H	4.52584800	-1.84163900	1.36830800
H	3.73640100	-2.48486200	-0.09715900
H	3.10925600	1.64806400	0.93837000
H	4.11358500	0.65080900	2.02556400
H	4.73615100	1.12867600	0.41818300

h1···(2 ^{i}PrOH)

C	-1.42170500	-1.49459600	0.02169200
C	-2.76807400	-1.53965400	0.32701700
C	-3.60547500	-0.43048100	0.09940600
C	-3.03974400	0.74040700	-0.44853800
C	-1.69814300	0.80207600	-0.75930700
C	-0.83204200	-0.31953800	-0.54115500
H	-0.76841800	-2.35075300	0.20374800
H	-3.19906700	-2.45028900	0.75500800
H	-3.68895200	1.60229300	-0.62409500
H	-1.25834400	1.70868800	-1.18228800
C	-5.02141500	-0.50285100	0.42493000
H	-5.34949100	-1.47933000	0.85549900
O	-5.83329700	0.39222600	0.26435400
O	0.41738200	-0.26981000	-0.83706900
H	1.26273700	-1.68197100	-0.70512900
O	1.84917800	-2.44182400	-0.45578400
C	2.89643400	-1.87446600	0.30213700
H	2.47428100	-1.28485700	1.14176100
C	3.72276200	-3.00825000	0.88282100
C	3.73748500	-0.93988700	-0.56084300
H	4.16164300	-3.60804700	0.06969500
H	4.53889300	-2.62602600	1.51278900
H	3.09162100	-3.67004400	1.49423900
H	3.12095500	-0.11884100	-0.95840700
H	4.56786100	-0.49928800	0.01222100
H	4.15888700	-1.49854000	-1.41202100
H	1.16350000	1.18446400	-0.99378500
O	1.67187400	2.03710500	-0.99467800
C	2.06406600	2.29985500	0.33619800
H	2.66106600	1.44917000	0.72409100
C	2.95150000	3.53195800	0.32066800

C	0.85698500	2.47974100	1.24884600
H	2.38801100	4.39572600	-0.06661100
H	3.31407000	3.77553300	1.32969400
H	3.82133300	3.36956500	-0.33292100
H	0.24189400	1.56696000	1.26173000
H	1.16943900	2.69342700	2.28228100
H	0.23119100	3.31469900	0.89469000

h1···(3 ^{i}PrOH)

C	-1.97534100	-1.08132900	-1.05975000
C	-3.35318800	-1.18561600	-0.99001100
C	-4.12739900	-0.17564300	-0.39229100
C	-3.47438100	0.95684100	0.13359200
C	-2.10042800	1.07361100	0.07146900
C	-1.29888500	0.05224700	-0.52364400
H	-1.37707400	-1.86307800	-1.53362000
H	-3.85520100	-2.06418000	-1.40665100
H	-4.07795600	1.74462300	0.59142400
H	-1.59225500	1.95494300	0.46953700
C	-5.57905000	-0.30539600	-0.32909100
H	-5.98351600	-1.24195400	-0.78172000
O	-6.33733200	0.50892400	0.16458800
O	-0.00796500	0.16168900	-0.57848400
H	0.96385000	-1.06452200	-1.21947400
O	1.62619400	-1.74233400	-1.49459500
C	2.90672900	-1.14806100	-1.39975800
H	3.11456700	-0.88464500	-0.34336800
C	3.92595000	-2.17986000	-1.84844200
C	2.97980600	0.12644200	-2.23159000
H	3.73682100	-2.46294000	-2.89615100
H	4.94994300	-1.78676400	-1.77174800
H	3.85533800	-3.08829500	-1.23164900
H	2.22440900	0.85489400	-1.89975400
H	3.96970900	0.60070500	-2.14491800
H	2.79362100	-0.10337100	-3.29317100
H	0.54387400	1.74341200	-0.66776700
O	0.96966300	2.63468400	-0.68811400
C	1.92253400	2.65431500	0.35680000
H	2.51320300	1.71647900	0.33556100
C	2.86044800	3.82173700	0.10605900
C	1.23898200	2.74822200	1.71541000
H	2.28969000	4.76402900	0.09076300
H	3.62820000	3.89174600	0.88995800
H	3.36386900	3.71022100	-0.86589800

H	0.56668900	1.89086000	1.86516900
H	1.97634400	2.74400200	2.53268100
H	0.64880100	3.67667400	1.77890400
H	0.98931700	-0.27713700	0.81996800
O	1.51418000	-0.47742800	1.62345900
C	1.50139800	-1.87416600	1.85462000
H	2.23654200	-2.36610600	1.18602000
C	1.93000000	-2.10127400	3.29300600
C	0.13154900	-2.46296500	1.55606500
H	1.20143300	-1.64375300	3.98085700
H	2.00132500	-3.17483700	3.51915700
H	2.91240000	-1.64187200	3.47722700
H	-0.10903400	-2.32797500	0.49205600
H	0.10717000	-3.54015300	1.77865000
H	-0.64234100	-1.95991100	2.15846500
